# Climate-induced variability in South Atlantic wave direction over the past three millennia

**DOI:** 10.1038/s41598-020-75265-5

**Published:** 2020-10-29

**Authors:** A. P. Silva, A. H. F. Klein, A. F. H. Fetter-Filho, C. J. Hein, F. J. Méndez, M. F. Broggio, C. Dalinghaus

**Affiliations:** 1 Graduate Program in Oceanography, Federal University of Santa Catarina, Box 476, Florianópolis, SC 88040-900 Brazil; 2 Griffith Centre for Coastal Management (GCCM), Building G51, Griffith University, Gold Coast, QLD 4215 Australia; 3 Virginia Institute of Marine Science, William & Mary, P.O. Box 1346, Gloucester Point, VA 23062 USA; 4 Department of Sciences and Techniques in Water and Environment, Cantabria University, Santander, Spain

**Keywords:** Palaeoceanography, Palaeoclimate, Ocean sciences, Physical oceanography

## Abstract

Through alteration of wave-generating atmospheric systems, global climate changes play a fundamental role in regional wave climate. However, long-term wave-climate cycles and their associated forcing mechanisms remain poorly constrained, in part due to a relative dearth of highly resolved archives. Here we use the morphology of former shorelines preserved in beach-foredune ridges (BFR) within a protected embayment to reconstruct changes in predominant wave directions in the Subtropical South Atlantic during the last ~ 3000 years. These analyses reveal multi-centennial cycles of oscillation in predominant wave direction in accordance with stronger (weaker) South Atlantic mid- to high-latitudes mean sea-level pressure gradient and zonal westerly winds, favouring wave generation zones in higher (lower) latitudes and consequent southerly (easterly) wave components. We identify the Southern Annular Mode as the primary climate driver responsible for these changes. Long-term variations in interhemispheric surface temperature anomalies coexist with oscillations in wave direction, which indicates the influence of temperature-driven atmospheric teleconnections on wave-generation cycles. These results provide a novel geomorphic proxy for paleoenvironmental reconstructions and present new insights into the role of global multi-decadal to multi-centennial climate variability in controlling coastal-ocean wave climate.

## Introduction

The predominant wind-wave climate has been shifting in both direction and magnitude^1–3^ in each the Northern^4^ and Southern Hemispheres^5–7^ in recent decades, a phenomenon likely in response to altered atmospheric dynamics and interhemispheric teleconnections in association with increasing global temperature^3,8–10^. Satellite-based measurements and reanalysis hindcast data reveal the presence of these changes over longer, historical periods^1–3,7^; however, even these only allow for exploration of the past 30–60 years of wave data, a time period marked by intensifying climatic changes. The extension of wave-climate analysis into prehistoric and recent geologic periods enhances the quantification of linkages between past trends and frequencies of climatic oscillations, as well as associated impacts on coastal-ocean wave variability^11^. This information supports insights into likely future shifts in wave climate given global climate-change projections.

Few studies have investigated paleo variability in wave climate^12–15^, largely because near-instantaneous signals such as wave height and period are difficult to derive from geological record. However, such archives may document changes in the long-term mean directional wave energy flux (EF_θ_), which represents the rate of total energy transported in an orthogonal direction to the wave-crest propagation over a period of time. Along embayed beaches, for instance, the mean wave energy flux is recorded in shoreline orientations with respect to the direction of the predominant wave climate reaching the coast^16–20^. Continued coastal progradation allows for the progressive removal of individual shore-parallel beach-foredune ridges (BFRs) from the profile active zone. Within this process, the beach geometry (planform and profile) is preserved over periods of centuries to millennia, over which coastal strandplains develop^12,13^. Because the variability of EF_θ_ reflects changes in the predominant wave-generating atmospheric systems, normally driven by large-scale climate drivers^3,5^, BFR sets are potential multi-decadal-scale proxies for the direction of predominant wave energy flux approaching the shoreline; and by extension, offshore wave climate and associated atmospheric variability over the adjacent ocean basin.

**Figure 1:**
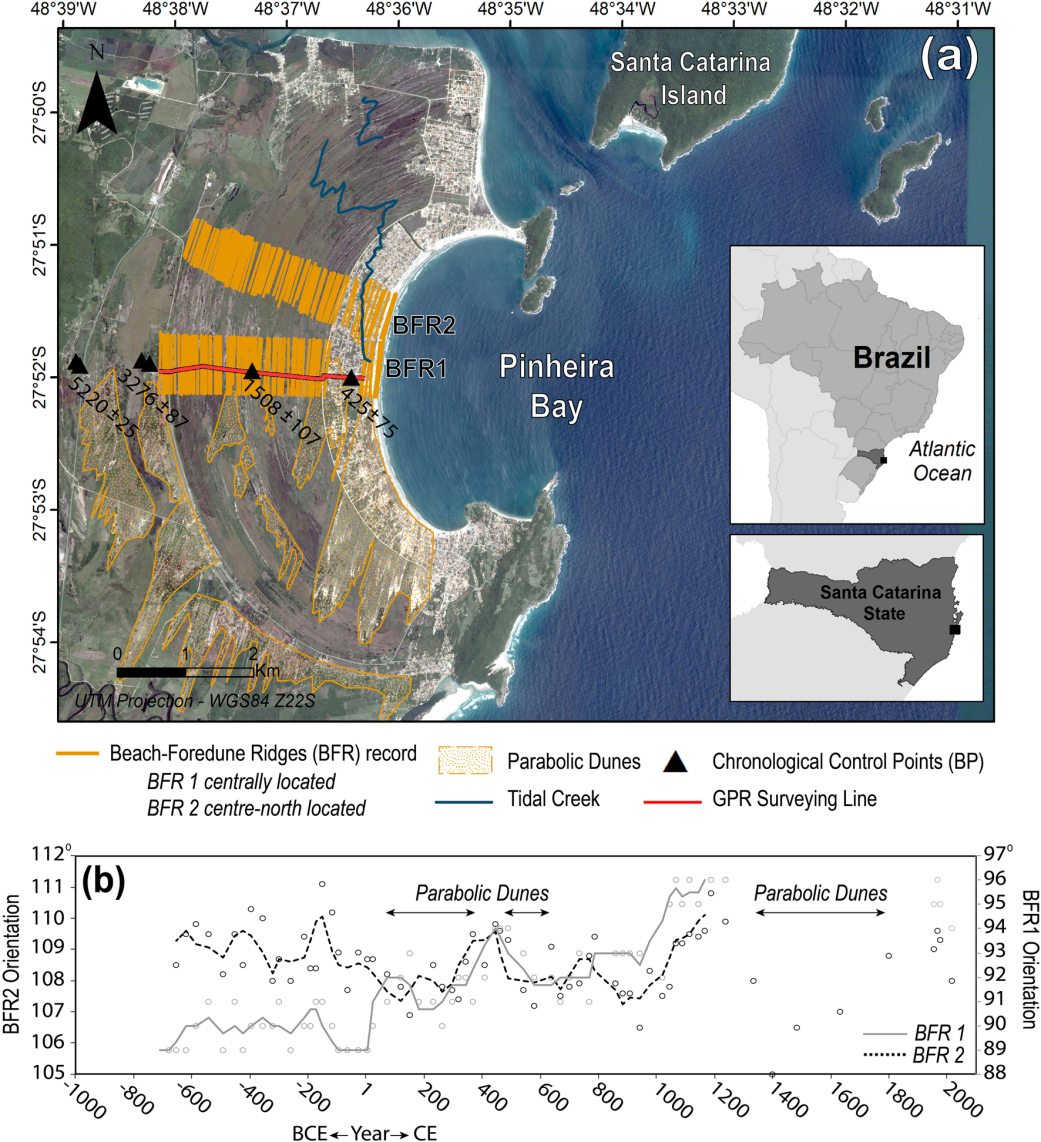
Beach-foredune ridges (BFR) records at Pinheira Strandplain, Brazil. (**a**) Location map of the Pinheira Strandplain (Santa Catarina, Brazil) highlighting mapped BFR1 (central plain) and BFR2 (north-central plain) sequences, and overlying parabolic dunes. Red transect indicates the location of the ground-penetrating radar survey line (Fig. SI1.2) and black triangles indicate geochronological control points (years Before Present; present = 1950), both derived from Hein et al.^32^. (**b**) The orientations of BFR1 (grey circles) and BFR2 (black circles) are plotted by approximate formation age (*CE* Common Era, positive values; *BCE* Before Common Era, negative values). Lines indicate the moving average (n = 3) for BFR1 (grey line) and BFR2 (black-dashed line). [Map created using ArcGIS Desktop 10.3—https://www.esri.com/en-us/arcgis; Image from Google, Maxar Technologies].

Here, we use a 3-km wide, semi-continuous sequence of BFR preserved within the Pinheira Strandplain (Santa Catarina State, Brazil) (Fig. 1) to map the geometry of former shorelines and derive a 3000-year record of inferred mean wave direction. Further, we explore connections between oscillations in the predominant wave direction and forcing mechanisms influencing the wave generation. In this manner, we use this morphology-based paleoclimate proxy to better constrain Late Holocene wave-climate variability within the understudied South Atlantic, and to support paleoceanographic studies by refining the relationship between leading atmospheric variability and long-term beach morphodynamics, extending those to protected, highly embayed, headland-dominated coasts.

## South American wave climate setting

**Figure 2:**
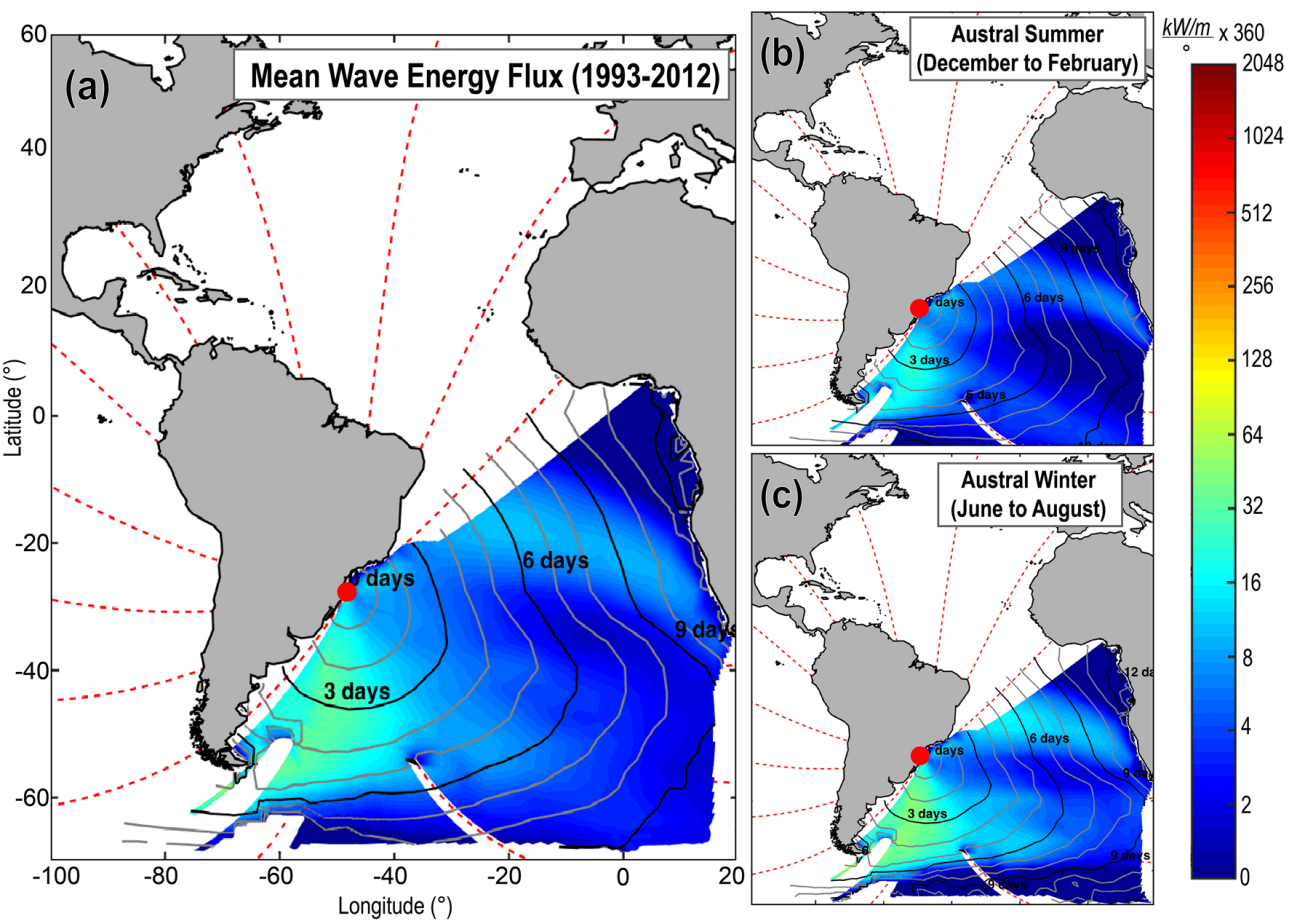
Mean wave-energy-flux generation zones. Twenty years of mean wave-energy fluxes are shown for (**a**) annual-averaged period; (**b**) mean austral summer; and (**c**) mean austral winter. The coloured area represents the spatial domain of the potential source regions. Warmer colours indicate the most energetic wave-generation areas capable of influencing the study area (red circle). [Maps create using the ESTELA method^26^ in Matlab Desktop R2018b—https://www.mathworks.com/].

Over recent decades, the Subtropical South Atlantic wave climate approaching the South American coast has presented a frequent bimodal sea-swell configuration^21–25^, with the east (E) to south-southeast (SSE) sectors representing 80% of the wave climate^7,10,21^. The mean effective energy flux^26^ reaching the study region has been largely influenced by waves generated in the mid- to high-latitudes (30–60°S and 40–70°W) of the South Atlantic basin (Fig. 2a). The high-energy SSE swells from this region are commonly generated by long and intense wind fetches resulting from extratropical cyclones^21–23^ migrating around the northern edge of the Southern Ocean, and sometimes moving northwards along the southern end of South America, deviating eastward into the South Atlantic around 35–40°S^27,28^. This component of the mean effective energy flux is observed throughout the year, but it dominates from the austral autumn to winter^21,22^ (Fig. 2c). During this season, the density of the cyclogeneses below 40°S prevails over lower latitudes^27,28^ while the intensification and longitudinal spread of the South Atlantic Subtropical High (SASH) also strengthens the sea-level pressure gradients and wind fields^28–30^ in higher latitudes.

The easterly wave component of the mean effective energy flux is locally generated (Fig. 2a) in response to the persistent north-easterly winds at the boundary of the semi-permanent SASH system^21,22,31^. A strengthening of the local wind fields tends to occur with the passage of a frontal systems, normally accompanied by high and low pressures systems that intensify the sea-level pressure gradients in the region^27,28^. These strong anti-cyclones can persist nearly stationary off the coast for several days, forming a long and persistent north-easterly wind fetch^21,27^. In this case, small easterly wind-seas progressively develop into larger waves with swell characteristics^22^. During summer, the bi-modal wave conditions are more frequent^21–23^ (Fig. 2b), while the southerly component is still present, but the easterly component is enhanced. In this season, the SASH migrates towards the centre of the South Atlantic basin^29^ creating NE-SW wind fields tangent to the coast^30,31^, and low-pressure systems are observed initiating or moving along the coast up to 20–25°S^27,28^, strengthening the local wave generation^21^.

## Results and discussion

### Wave-direction proxy record

Shoreline morphology evolves in response to a complex suite of short- and long-term morphodynamics agents acting in the coastal zone. In exposed beach-embayments, these includes changes in regional longshore sediment transport and related processes such as headland or river-mouth bypassing as well as the frequent temporal and spatial variations in bathymetry^19^. In contrast, within sheltered embayments, particularly those lacking direct fluvial sediment inputs, sediment is contained within the beach compartment and transport becomes predominantly cross-shore^18,19^. Here, the beach planform tends to be adjusted towards a state of static equilibrium by aligning the downcoast orthogonally to the predominant wave fronts, following the premises of the Parabolic Bay Shape equation^16–18^. Shifts in shoreline orientation within a sheltered embayment require large and long-term changes in the predominant wave direction, in order to disturb the beach state towards a temporary dynamic equilibrium—when the shore-oblique wave breaking tend to induce sediment transport alongshore within the embayment until the downcoast adjusts orthogonally to the resultant wave front^17^. Therefore, the resulting preserved shorelines filter high-frequency wave variability and are more likely than those along open-coasts to preserve within the BFR orientations only the predominant multi-decadal mean directional wave energy flux.

Pinheira Strandplain was built during the last *ca.* 5000 years^32^ into a headland bay beach with two well-defined diffraction points, accentuated shadow zones, and a straight-central alignment that characterizes the downcoast^18^ (Fig. 1a). In the last ~ 2700 years, shorelines were preserved as a quasi-continuous sequence of 74 visible BFRs. Radiocarbon dating of molluscs derived from paleo-shorefaces^32^ (Fig. 1a) reveals linear progradation rates of 0.8–1.3 m‧year^−1^ which were used to estimate the formation age of individual BFRs. On average, growth and stabilization for each ridge required 20–30 years, indicating that these features preserve multi-decadal coastal processes. Any post-depositional modification of BFR morphology is expected to impact only the height or continuity of individual ridges, as opposed to the net alongshore ridge orientation.

**Figure 3:**
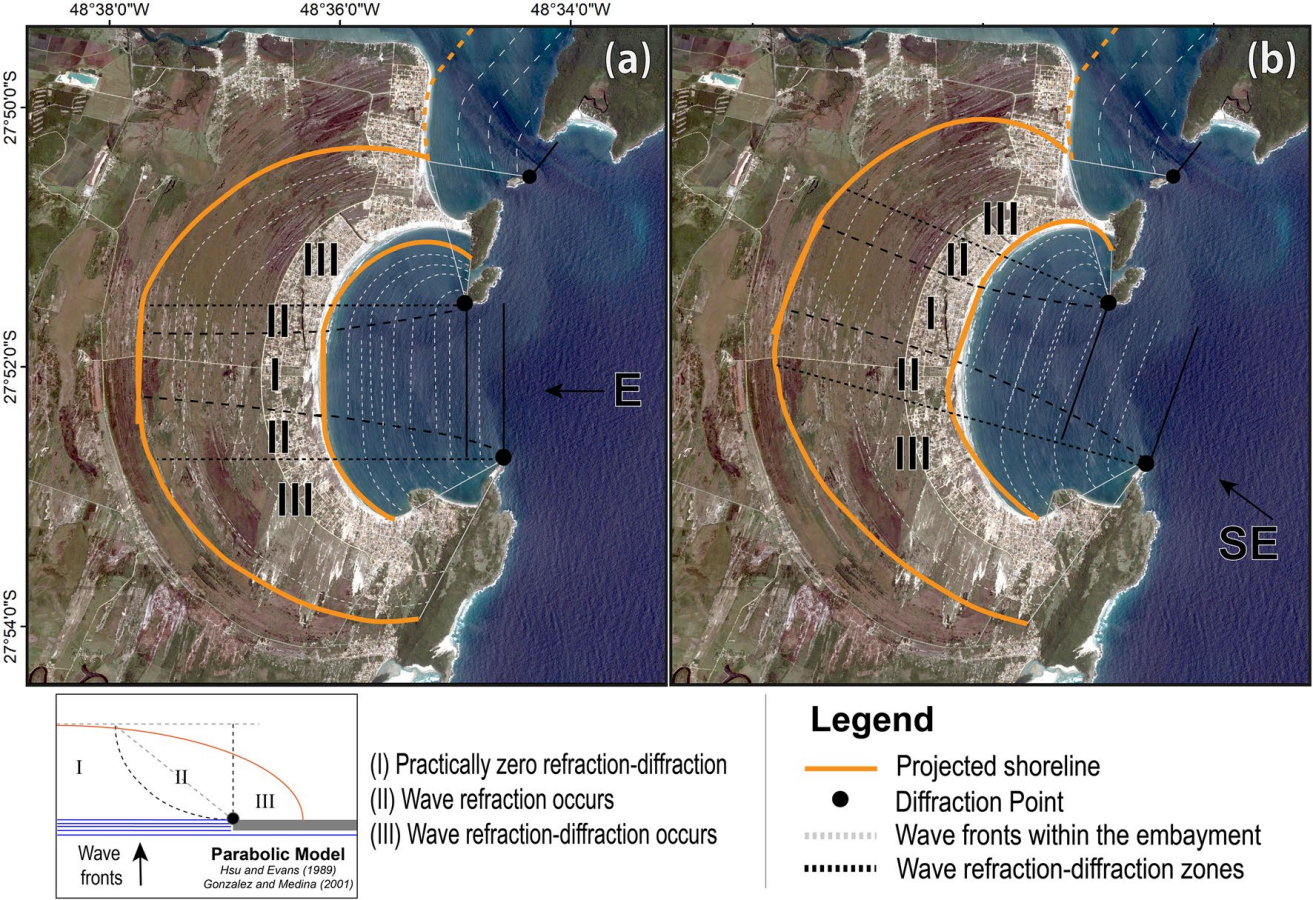
Equilibrium Beach Planform^18^ projections for modern and paleo-shoreline. (**a**) Equilibrium Beach Planform projected to predominant easterly wave fronts reaching the diffraction points. (**b**) Equilibrium Beach Planform projected to predominant southeasterly wave fronts reaching the diffraction points. Orange lines indicate the projected shoreline; dashed grey lines indicate the wave fronts propagation within the embayment; black dashed lines define the wave transformation zones (I, II, and III) according to the parabolic bay equation parameters^18^. [Projections were estimated using the SMC-Brazil long-term analysis module^39^ and vectorized using Adobe Illustrator CC 2018. Background map created using software ArcGIS Desktop 10.3; Image from Google, Maxar Technologies].

The rocky headlands fronting Pinheira situate the embayment into an enclosed cell considered in a state of static equilibrium (Fig. 3), with negligible inputs of sand derived from the regional littoral transport system. Modern bay bathymetry is smooth and seaward-deepening, characteristics also observed in chronosurfaces within the shallow terrestrial (see Supplementary Fig. SI1.2) and sub-bottom stratigraphy within central Pinheira Strandplain and Bay, respectively^32–34^. Furthermore, beach sedimentology—a determining factor of the long-term average profile shape^35^—is quasi-homogeneous (D_50_ = 0.16–0.2 mm) across the plain^32,34^. Given this, we assume a long-term equilibrium profile during the formation of the preserved BFRs until today, implying that shoreline has been dictated geometrically by comparable diffraction zones and shoreface refraction of the most persistent EF_θ_^18,19^ over the past few millennia.

The selection of the appropriate BFR sequences within Pinheira that could provide the paleo-wave records was projected (Fig. 3) by applying the parabolic bay shape equation^17,18^ that considers the shoreline stability as a function of the wave direction and the extension of the influence of the diffraction point to the downcoast. Based on this, we find that all sectors of the shoreline are subject to wave refraction processes for offshore EF_θ_ values of 45° (northeast; NE) to 180° (south; S). However, sensitivity to wave climate varies alongshore. Specifically, three zones are formed as the wave front enters the bay: (I) where the wave height gradients are practically zero and the wave fronts remain invariable, then (II) wave height gradients are formed and the waves suffer only the effect of refraction, and finally (III) where wave height gradients and the rotation of the fronts due to wave refraction–diffraction prevails^18^. Both the southern and northern ends of the strandplain are mainly within Zone III (Fig. 3), and thus associated shoreline orientations are assumed to be highly influenced by refraction-diffraction processes. Additionally, broad parabolic dunefields in southern Pinheira^36,37^ (Fig. 1a) and truncations of BFRs in the northern end of the strandplain^32^ (likely resulting from hydrodynamics associated with the surrounding bay entrance and tombolo formation), make these unsuitable for reconstructing offshore wave approaches.

In contrast, the central portion of the plain is within Zones I and II of various wave approach angles, and sensitive to offshore wave directions (E-SSE) that represent 91% of the variability at the deep-water (111 m) wave buoy (see Supplementary Table SI2.1). We thus select two sub-parallel BFR sequences (Fig. 1a) for paleo-wave analysis. BFR1 is located within the central plain, oriented orthogonally to the easterly wave fronts (Fig. 3a), and subject to the highest wave energy flux approaching from ~ 85° to 150° of offshore direction (93°–125° at the entrance) (see Supplementary Figs. SI2.2, SI2.3a). BFR2 is located along the north-central portion of the plain, is orthogonally oriented towards southeasterly wave fronts (Fig. 3b), and is subject to the highest wave energy flux approaching from ~ 90° to 180° of offshore direction (96° to 133° at the entrance) (see Supplementary Figs. SI2.2, SI2.3b). This approach neglects wave incident from the northeast (incoming angle of 45°–75°). However, at 60 m depth—offshore of where they interact with any coastal features—waves from this sector present a mean wave energy flux of only ~ 50 to 155 J‧s^−1^‧m^−1^ (see Supplementary Fig. SI2.4), a range incapable of effective morphological changes^20^. In addition, the signal of high-energy NE swells typically generated by tropical cyclones are absent at latitudes higher than 20–25°S along the South American coast^21,22^, since these atmospheric systems rarely move onto the Subtropical South Atlantic even under the air-surface temperature warming during the last decades^38^. For this reason, the BFRs located within the south-central portion of the strandplain, which are oriented orthogonally towards the NE wave fronts and largely covered by parabolic dunes, were omitted from analysis.

Our approach to reconstructing offshore wave climate from BFR orientations is supported through application of a wave-propagation model to a single paleoshoreline dating to ~ 170 Before Common Era (BCE), reconstructed from stratigraphic analyses (see Supplementary Information—Section 1). The results revealed considerable agreement with the observed modern wave-transformation processes within the bay, with the exception of waves from the NE sector. At present, these NE waves retain influence of diffraction around the northern headland as they approach the modern shoreline. However, in the past (such as at 170 BCE), NE waves refracted along the longer profile after entering the embayment, becoming more shore-normal oriented than at present (see Supplementary Fig. SI2.1).

The BFRs analysis reveal that shoreline evolution occurred in multi-centennial cycles ranging from ~ 89° to 96° for sequence BFR1 and ~ 105° to 111° for sequence BFR2 (Fig. 1b). To attribute each degree of shoreline rotation to a specific offshore wave direction, we applied a well-resolved diffraction-refraction numerical model (SMC-Brazil)^39^ that simulated wave propagation in 5° bins, ranging from 45° to 180°. Results showed that the highest wave energy flux approaching from 85° to 150° reach the BFR1 section of the plain with EF_θ_ of 89° to 95°, while for BFR2, offshore waves from 90° to 180° approach with EF_θ_ of 104° to 114° (see Supplementary Fig. SI2.3a,b). Therefore, we find BFR orientations show correspondence with the EF_θ_ of waves reaching 5 m water depth, prior to wave breaking; this allows for correlation of shoreline orientations with offshore wave direction.

### Late holocene wave direction and atmospheric patterns variability

**Figure 4:**
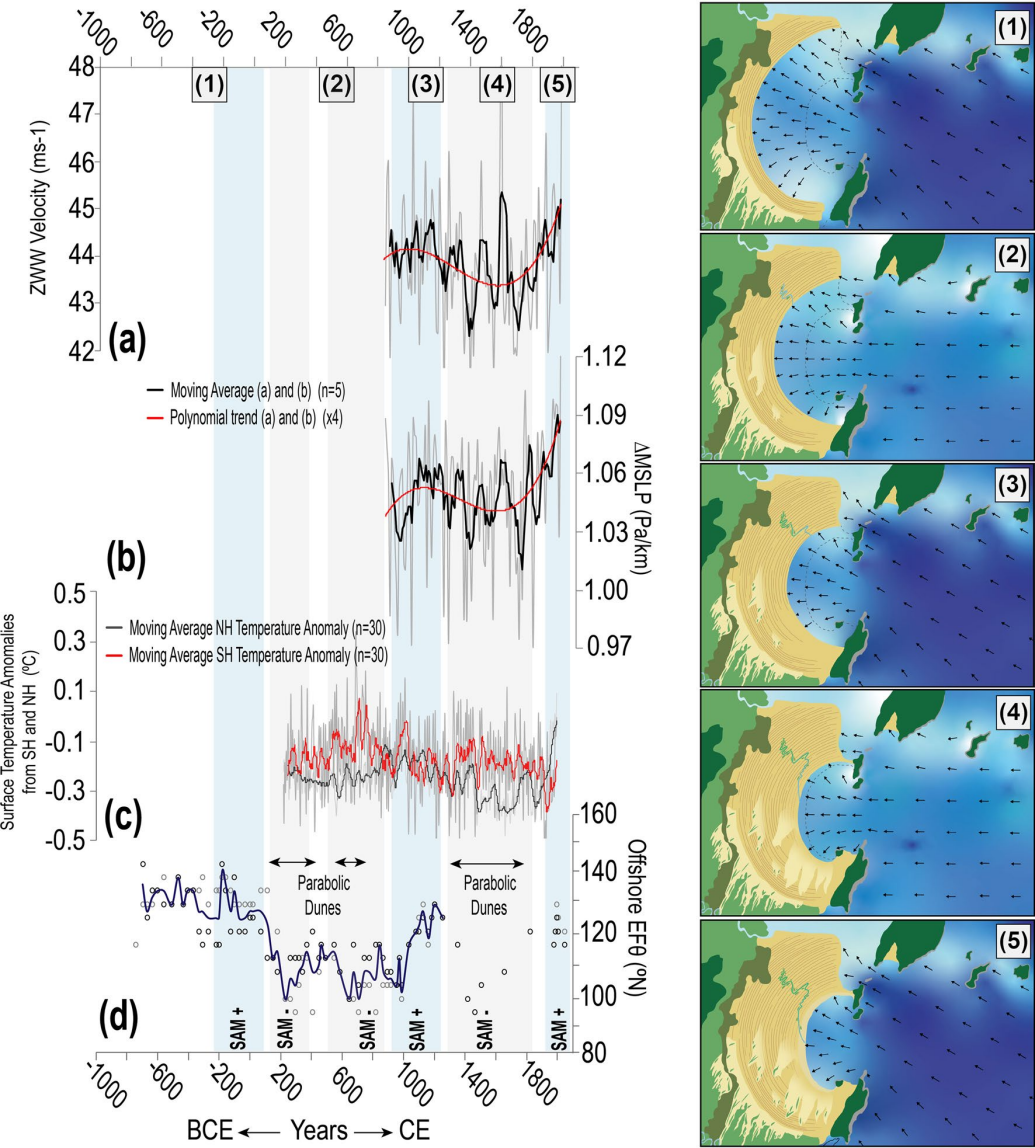
Proxy records of offshore mean wave direction and climate forcings. Decadal averages of (**a**) zonal westerly winds (ZWW) velocities^42^ and (**b**) mid-latitude gradients in mean pressure at sea level (ΔMSLP)^42^; black line represents the moving average (n = 5), and red line is the polynomial trend (× 4). (**c**) The annual variability of surface air temperature anomalies for the SH and NH^51^ are presented accompanied by the moving average (n = 30); red line indicates the SH and grey line the NH. (**d**) The offshore mean wave direction is presented for the last 2700 years; circles indicate the estimated wave direction from the BFR sequences while the dark blue line represents the mean value between BFR1 and BFR2. Periods of Southern Annular Mode (SAM)^46^ anomalies are indicated as well as periods of parabolic dune formation at Pinheira. Finally, the strandplain evolution under the different wave directions is exemplified in a schematic map for each phase (1, 2, 3, 4 and 5). The blue gradient indicates wave height and the black arrows indicate wave direction based on the wave propagation model results (see Supplementary Information—Section 3). [Schematic maps created using Adobe Illustrator CC 2018—https://www.adobe.com/pt/products/illustrator].

Causes of variability in the Subtropical South Atlantic offshore EF_θ_ over last ~ 3000 years were inferred from a combination of variations in the modern mean effective wave energy flux and the potential atmospheric and climate patterns known to influence the multi-decadal shifts in the predominant wave climate. The oldest 25 ridges within our continuous Pinheira sequences formed between ~ 700 BCE and 100 CE (Fig. 4, Phase 1). Paleoshorelines orientations from this period vary between 89° and 91° within transect BFR1, and 108° and 111° within transect BFR2 (Fig. 1b). The northern end of the plain was marked by considerable dynamism due to the connection with the adjacent bay. It is therefore presumed that high-energy swells from the SSE (Fig. 4d), most likely corresponding to offshore EF_θ_ > 130° (see Supplementary Information—Fig. SI2.3), would have persisted during this period promoting the sediment deposition along the northern region of the strandplain. The predominance of such SSE swells might have been a product of an intensification of associated mid- to high- latitude wave-generation zones (Fig. 2).

In the following centuries, between ~ 200 and 900 CE, BFR1 shoreline alignments generally varied from 90 to 93° with a peak of 94° around 400–500 CE. This is in agreement with shoreline orientations within BFR2, which varied between 107° and 109°, peaking at 110° in the same period. These orientations are interpreted as a persistent ESE (90° to 120°–125°) offshore EF_θ_ (Fig. 4d, Phase 2). During this phase, BFRs were formed coinciding with the development of sets of NE-to-SW-migrating parabolic dunes that cover the southern portion of the strandplain^36,37^. The development of parabolic dunes suggests sand availability through the rapid reworking of beach and foredune sediment by intense and frequent NE winds^36,40^. A decrease in wind velocity and direction can cause stabilization of the dunefield, due to reduced sediment transport and consequent vegetation establishment^40^. Therefore, periods marked by development of parabolic dune fields would require relatively strong orthogonally oriented wave energy that would reach the beach foreshore and backshore along the shoreline periphery, resulting in unstable frontal dunes^40^ and sand mobilization primarily through cross-shore transport. Such conditions require the strengthening of the local wind fields and wave generation zones (Fig. 2), which today is likely associated with intense transient anti-cyclones following the passage of low-pressure systems^27,28^. These systems tend to have stronger easterly wave component as a resultant of the locally generated energy flux^21,22^ (Fig. 2). Moreover, a persistent influence of the NE winds generated by the offshore west branch of the SASH^30,31^ would maintain the active dunefield. Transgressive dunefields are also reported for the Cassino Strandplain (Rio Grande do Sul, Brazil) for the periods between ~ 100 and 500 CE and ~ 1750 CE^41^, suggesting that the formation of dunefields in both locations—located 700 km apart—has been controlled by the variability of large-scale atmospheric patterns and teleconnections.

**Figure 5:**
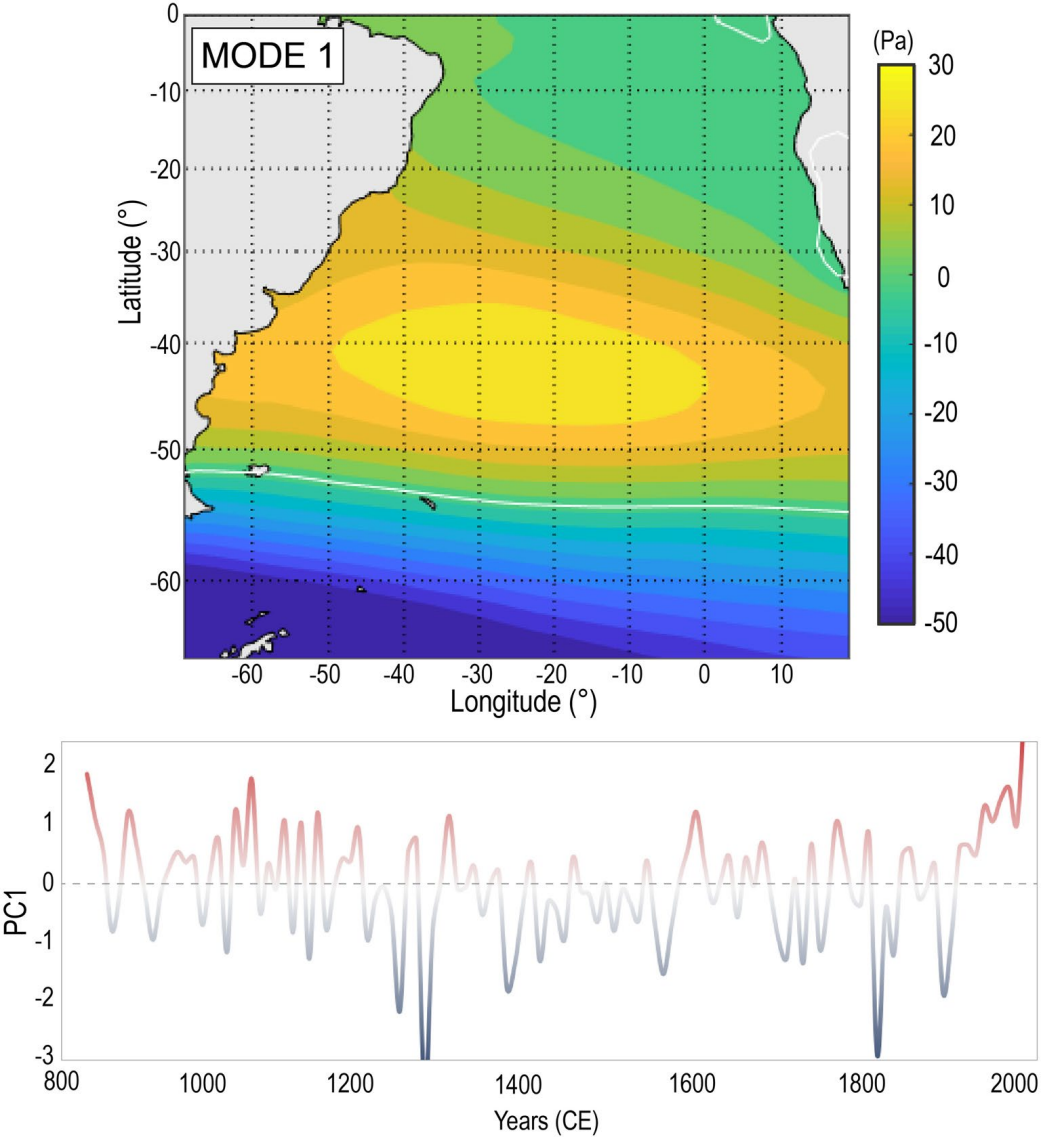
Leading EOF mode (43%) of decadal sea-level pressure variability over the South Atlantic basin between 800 and 2000 CE. Empirical Orthogonal Function (EOF) of sea level pressure (Pa), above, and the corresponding principal component time series. This analysis used sea level pressure time series from the CESM1-CAM5 “Last Millennium Ensemble (LME)”^42^. [Map created using Matlab Desktop R2018b].

From 850 CE until present, we investigate the variability in wave direction in comparison to decadal means of mid- to high-latitudes mean sea-level pressure gradients (∆MSLP) and zonal westerly wind velocities estimated from the CESM1-CAM5 “Last Millennium Ensemble (LME)”^42^. A principal component analysis of the mean sea level pressure on the South Atlantic over decadal scale identified six leading Empirical Orthogonal Functions (EOF) modes explaining 92% of the variability (see Supplementary Information Fig. SI4.1), with 43% being attributed to the first mode alone (Fig. 5). Thus, we focused here on the influence of the leading EOF mode 1 on the Subtropical South Atlantic wave direction variability during this last millennium, with particular emphasis on multi-centennial cycles responsible for large wave direction shifts influencing the study area. We note that while we focus on this singular mode in this study, wave generation and climate in the Subtropical South Atlantic results from variability in a number of atmospheric patterns, large-scale drivers and teleconnections^3,7^, many of which remain understudied as compared with other regions globally.

Between 850 and 1200 CE, EOF 1 resembles the variability of the Southern Annular Mode (SAM) (Fig. 5) shifting towards its positive phase^43,44^. Positive SAM phases consist of a clear strong atmospheric pressure gradient around 50°S, fringing the Southern Ocean border, formed in response to a well-defined South Atlantic High over the Subtropical South Atlantic^43^, and coupled with a quasi-stationary low pressure in high latitudes (Fig. 5). This condition tends to increase the zonal westerly wind velocities, as observed in Fig. 4a, and decrease the intensity of surface zonal wind fields in lower latitudes^44^. Previous studies of SAM variability in the past few millennia^44–48^ also report similar temporal variations based on a variety of paleoclimate proxies. In terms of wave response, between 1000 and 1200 CE, a southerly realignment occurred at both sequences BFR1 and BFR2 (Fig. 1b), which is indicative of a clockwise shift to a SE (120°–135°) offshore EF_θ_ (Fig. 4d, Phase 3). Thus, positive SAM is attributed to the strengthening of the higher latitude wave generation zones (Fig. 2), favouring a most persistent south-southeast swell.

At 1200–1300 CE, a transition towards the negative phase of EOF 1 is observed (Fig. 5), coinciding with that detected in the Southern Indo-Pacific, in which atmospheric patterns moved from an expanded Hadley Cell phase (1000–1220 CE) and strong atmospheric blocking over southern South America (1150–1220 CE) toward an equatorward-subtropical ridge and stronger high pressure over Antarctica (1300–1350 CE)^49^. Such negative SAM phases are also characterized by northerly displaced subtropical jets, increasing cyclogenetic activity in latitudes lower than 40°S, near the South American coast^43^. With a weakening of the ∆MSLP in higher latitudes (Fig. 4a,b), negative-phased SAM reduces the relative contribution of higher-latitude wave-generation zones and favours the increase of the locally generated wave energy flux (Fig. 2). This phase persisted until ~ 1600 CE, returning to an average SAM with two strong peaks towards the negative phase again between ~ 1800–1900 CE (Fig. 5). At Pinheira, BFR2 shorelines presented an anticlockwise shift (105°–108°) from ~ 1200 to 1600 CE (Fig. 4d, Phase 4), which indicates an active easterly wave component. By 1800 CE (Fig. 1b), BFR2 was shifting back towards SE. Shoreline records at sequence BFR1 are obscured by super-imposed parabolic dunes during this period. Extensive parabolic dunefields were formed over last 800 years, possibly under more than one stage of development^36,37^. Although the complex combination of factors that control the parabolic dunes dynamics from the initiation to stabilization are not fully investigated here, we suggest that the increased storminess in lower latitudes influenced by negative SAM phases^43^ would have supported the backshore instability and the occurrence of strong NE winds carrying sand from the beach, burying the developing vegetation and activating the mobile dune system development^40^.

From ~ 1900 CE until present, EOF 1 shifted to a strong positive phase (Fig. 5), in agreement with the strengthening of ∆MSLP and zonal westerly wind velocities in mid- to high-latitudes of the South Atlantic (Fig. 4a,b). Several studies of SAM variability have reported a continued trend towards a positive phase^44,45,48,50^, one which is unprecedent in amplitude as compared with oscillations reconstructed for the last few millennia^44–48^. In this sense, the twentieth century trends towards positive SAM are likely to induce a clockwise shift of wave direction due to intensified mid- to high-latitude wave generation zones in Subtropical South Atlantic, which has been suggested by previous studies^3,5,7^. Along South American coast, a spatial correlation (0.2 to 0.3, p < 0.05) between SAM and a 60-year (1948–2008) monthly EF_θ_^7^ suggests the increasing of the southerly swell component within positive SAM phases. Additionally, a positive correlation between SAM and 60-year (1948–2008) annual offshore mean energy flux (0.4, p < 0.05) was obtained for the wave reanalysis data used in this study, and similar result was also reported for significant wave height (0.5, p < 0.05) within a 30-year (1979–2010) analysis of the wave conditions in the vicinity of the study area^24^. These correlations are likely the strongest links detected up to date between a large-scale climate driver and wave climate on the Subtropical South Atlantic^3,7^. In response to these trends, a southeast-ward shift in average foredune-shoreline alignments at Pinheira is predicted (Figs. 1b, 4d, Phase 5).

This newly established relationship between large-scale atmospheric circulation, wave direction in Subtropical South Atlantic, and the orientation of preserved BFR allows for records of multi-decadal SAM variability (as expressed through offshore EF_θ_) to be extended beyond the period covered by the LME reanalysis series. For example, between ~ 200 and 900 CE, easterly wave predominance (Fig. 4d) recorded in BFR orientations implies a higher frequency of negative SAM, with a potential short positive phase centred ~ 400–500 CE when a rapid clockwise shift in shoreline alignment occurred (Fig. 1b). In contrast, prevailing southeasterly swells between 700 BCE and 100 CE (Fig. 4d) are associated with longer periods of positive SAM. These findings are largely consistent with those from the longest-available (*ca.* 3000 years) SAM proxy reconstructions, derived from pollen and charcoal in sediments from Lake Cipreses (Chilean Patagonia)^46^. However, results diverge for the first 300 years of the paleo-sequence, when a humid/cold phase suggested a negative SAM stage from the Chilean records, whereas BFRs retain an orientation consistent with SSE wave predominance associated with a positive SAM phase. This disagreement may stem from the geographic location of the paleo-records and the sensitivity to the atmospheric changes incurred from the SAM anomalies in that period, or even the interference from other climate drivers conditioning some divergence in the anticipated atmospheric patterns and proxy responses.

### Insights into climate impacts on subtropical South Atlantic wave direction

Although the precise forcings—solar irradiance, greenhouse gas concentrations, stratospheric ozone depletion, internal dynamics, etc.—remain under debate, centennial-scale climate anomalies over last millennia (e.g. Medieval Warm Period (~ 800–1300 CE) and Little Ice Age (~ 1350–1850 CE), as well as during twentieth century warming)^51^ have been observed to coexist with atmospheric patterns variations in both hemispheres, either in low-latitude systems such as the Intertropical Convergence Zone^52,53^ or in mid- to high-latitudes atmospheric modes such as the Southern Annular Mode^44,46^. Consequently, wave conditions are expected to respond to these climatic variations. Over recent decades, for example, positive correlations were obtained between sea surface temperatures in the tropical and North Atlantic and wave power in the southern extra-tropics, suggesting intensification of the transfer of energy from the wind fields into wave generation in these regions as a result of the warming trends^3^.

We compared the variability of the offshore EF_θ_ derived from the BFRs for the period between ~ 200 and 1950 CE with the surface air temperature anomalies^51^ in both hemispheres (Fig. 4c,d), obtaining significant negative correlation for the Southern (− 0.4, p < 0.01, n = 41) and positive for the Northern (0.25, p < 0.01, n = 41) hemisphere. The opposite signal for these correlations might occur as a result of these surface air temperature excursions being non-uniform across both hemispheres (Fig. 4c)^51,54–56^. Specifically, the results suggest that periods of relatively warmer Southern Hemisphere as compared with Northern Hemisphere (e.g., during ~ 200–800 CE and the Little Ice Age) favours the predominance of easterly wave energy flux, whereas periods with equivalent temperature anomalies between both hemispheres (e.g., Medieval Warm Period) or those in which the Southern Hemisphere was relatively colder (last ~ 150 years), support an increase in the influence of the southerly wave energy flux along the eastern South American coast. In fact, this interpretation describes a similar pattern to the current seasonal wave energy flux variability in the Subtropical South Atlantic (Fig. 2b,c). Although the mechanisms governing these correspondent multi-centennial shifts in wave direction and paleoclimate are currently unidentified, these results imply on climatic changes inducing long-term wave-climate variability in the South Atlantic, and by extension, deriving regional and local changes in coastal morphodynamics.

## Conclusion

Our data reveal that climate changes over the Late Holocene induced modifications to wave conditions at the scale of an ocean sub-basin. This suggests a likelihood of substantial future shifts in wave climate in response to forecasted twenty-first century climate change. The response of waves to climate-change-associated atmospheric variability differs across ocean basins^2,3^, highlighting the need for studies of long-term changes in wave climate at the regional-scale; our new approach developed here, relying on wave-energy and wave-direction changes preserved in the paleo BFR orientations, presents a tool for these reconstructions.

The BFR analysis indicated multi-centennial oscillation of the Subtropical South Atlantic wave direction that respond to large-scale atmospheric drivers and climate anomalies. Here we identified the Southern Annular Mode as a leading climate driver inducing the wave generation in mid- to high-latitudes and consequently, influencing the relative contribution of the high-energy southerly swells to the mean effective wave energy flux propagating throughout the basin, particularly along the South American coast. The extension of this analysis throughout the eastern South Atlantic coast and tropical regions is recommended for future studies, as it would add to a detailed understanding of the long-term wave direction variability over the whole ocean basin and contribute to evaluate the influence of other atmospheric patterns driving the wave climate in the region. Given the central role of wave climate in controlling littoral transport dynamics, long-term shifts in wave direction alter the movement of sediment within and across beach compartments respective to their degree of exposure to the approaching swell. Understanding and better forecasting these changes is fundamental for future coastal adaptation and management.

## Methods

### Beach-foredune ridge (BFR) record

In order to identify the shoreline sectors that are orthogonally oriented to the predominant wave fronts reaching the embayment, an equilibrium beach planform projection was applied using the long-term analysis module of SMC-Brazil^39^. This approach characterizes the shoreline geometries that represent the static equilibrium^17,18^ of the embayment in response to the predominant mean directional wave energy flux (EF_θ_) arriving at the diffraction point at a given time through the application of the Parabolic Bay Shape Equation. The results were vectorized in a graphic software and presented in Fig. 2.

Following selection of two cross-shore BFR transects, the historic BFR orientations were mapped within a geographic information system through photointerpretation of an infrared orthophotography from 2010 (pixel size 0.4 m). Polylines of ~ 700 m length were traced tangent to the BFRs front, and true azimuth angles (given as 90° from shoreline orientation at all times) were calculated from each tangent. The error of the measurement is associated with the position of the starting and end points that can vary by ± 0.4 m at each end of the tangent due to the raster cell size, resulting in a BFR orientation error from the order of ± 0.001°, considered negligible. Aerial photographs from 1938, 1957, 1978 and satellite images from 2003, 2006, 2009, 2010, and 2012–2017 were rectified (RMS95% 6.7 m) and the orientation of the base of foredune vegetation was mapped on each. The approximate age of individual BFR was obtained through linear interpolation of strandplain progradation rates derived from ^14^C dates of molluscs across the plain; original sampling scheme and control-point geochronology is presented by Hein et al.^32^.

### Mean directional wave energy flux analysis

Wave propagation from deep to shallow water was simulated for 5° bins for offshore waves originating from 45 to 180 compass degrees. Numerical modelling of wave propagation was performed using the SMC-Brazil model, a non-dispersive spectral model that solves the wave phase, including refraction-diffraction processes for shallow water, through the parabolic approximation of the mild-slope equation^39^. The model was run with four general propagation grids (100 × 100 m resolution) and four nested grids (25 × 25 m resolution) with 30° of difference between their central axes (60°, 90°, 120° and 150°), covering the adjacent continental shelf to ~ 110 m water depth. Bathymetric data is provided by the wave model from digitized Brazilian nautical charts and GEBCO (General Bathymetric Charts of the Oceans).

The calibrated and validated Downscaled Ocean Wave (DOW) series consisting of 60 years (1948–2008) of reanalysis wave data at hourly resolution^25,39,57^ was obtained from a virtual buoy (DOW point; 27.8°S, 41.9°W) selected at 111 m depth, proximal to the shelf-break (130 m). From the data series, 100 representative cases were selected using the Maxima Dissimilarity technique^58^ for each 5° directional interval. Following wave propagation, the mean directional wave energy flux (EF_θ_) was reconstructed at the 5 m depth contour in central Pinheira Bay. This was done through an interpolation of the hourly, 60-year wave-climate data series based on the 100 cases propagated, using the Gaussian Radial Basis Function technique (RBF)^39,58^.

### Wave generation zones variability in South Atlantic

In order to identify the wave energy generation zones that influence the mean wave energy flux approaching the study area, the ESTELA (*Evaluating the Source and Travel-time of the wave Energy reaching a Local Area*) method^26^ was applied. A target point was chosen offshore from the coastal islands and headlands interference on the study area. Then a relevant spatial domain is delimited applying the geographic criteria which relies on the theory that deep-water waves travel in great circle paths. Following this, 20 years (1993–2012) of frequency–direction information is derived from spectral reconstructions using the WAVEWATCH III version 4.04 hindcast series, including parameters such as significant wave height, peak period, mean direction and directional spread for up to six partitions of the spectrum (the wind sea and five swell trains in the more general case). Finally, the effective wave energy is obtained considering the energy of the spectrum travelling to the target point at the group velocity, and corrected by the viscous dissipation that waves are expected to suffer during propagation from the source location to the target point. A detailed explanation of the ESTELA method is provided in Perez et al.^26^.

The paleo-climatic conditions influencing the variability of the predominant directional wave energy flux are analysed using the mean sea-level pressure gradient and zonal westerly wind speed from the Community Earth System Model—Last Millennium Ensemble (CESM-LME) reanalysis data series^42^. The ensemble members span ~ 1000 years, from 850 to 2005 CE, in monthly averages, and were simulated in full-forcing (solar radiation, greenhouse gases, aerosols, volcanic emissions gases, land use and orbital parameters)^42^. The only difference between each ensemble is a small random round-off (in the order of 10^−14^ °C) in air temperature at the start of each simulation^42^. In this study, atmospheric parameters were analysed based on the decadal-scale 13-ensemble average (115 decades), limited to the South Atlantic (10°N–90°S, 22.5°E–90°W), with an emphasis on middle to high latitudes. From this dataset, a principal component analysis was also applied, identifying the leading EOF modes for the South Atlantic sea-level pressure systems and their decadal-scale variability. A Pearson linear correlation was applied to the 2000-year surface air temperature anomaly curve proposed by Mann and Jones^51^ for the southern and northern hemispheres in relation to paleo offshore mean wave direction. In addition, linear correlations were also tested between the SAM^59^ index and the 60-year (1948–2008) annual wave energy flux from the virtual wave buoy. The statistical significance for all variables was examined through a *t* test at 95% level.

## Supplementary information

Supplementary Information.

## Data Availability

The data generated and analysed during this study are included in this published article and its Supplementary Information files. Supplementary datasets are available from the corresponding author on reasonable request.
