# Supplementary material for: Climate-induced variability in South Atlantic wave direction over the past three millennia
Source: Sci Rep. 2020 Oct 29;10:18553. doi: 10.1038/s41598-020-75265-5 (PMC7596712; doi:10.1038/s41598-020-75265-5)
Supplement: Supplementary file 1 — Supplementary Information. [file 41598_2020_75265_MOESM1_ESM.pdf]

CLIMATE-INDUCED VARIABILITY IN SOUTH ATLANTIC WAVE DIRECTION OVER PAST THREE MILLENNIA

SILVA, A.P.[a,b*], KLEIN, A.H.F.[a], FETTER FILHO, A.F.H.[a], HEIN, C.J.[c], MÉNDEZ, F.J.[d], BROGGIO, M.F.[a], DALINGHAUS, C.[a]

[a] Graduate Program in Oceanography, Federal University of Santa Catarina, Florianópolis, SC - 88.040-900, Brazil. [b] Griffith Centre for Coastal Management (GCCM), Building G51, Griffith University, Gold Coast Campus, QLD 4222, Australia. [c] Virginia Institute of Marine Science, William & Mary, P.O. Box 1346, Gloucester Point, VA 23062, USA. [d] Department of Sciences and Techniques in Water and Environment, Cantabria University, Santander, Spain

*Corresponding author address: M.Sc. Ana Paula da Silva
Griffith Centre for Coastal Management (GCCM), Building G51, Griffith University, Gold Coast Campus, QLD 4215, Australia/ Email: ana.dasilva@griffithuni.edu.au / Office: + 61 7 555 27834

SUPPLEMENTARY INFORMATION

This supplementary information presents detailed material on the reconstruction of the paleo-environments in Section 1, followed by the analysis of the mean directional wave energy flux from deep to shallow water in Section 2, the wave propagation charts in Section 3 and the leading EOF modes of sea-level pressure during last millennium in Section 4.

Section 1: Paleo-Environmental Record

Extension of the historic wave analyses and modelling as applied to the current coastline to the paleorecord provided by the preserved beach-foredune ridges (BFR) required the verification of changes the wave direction in response to changes in bathymetry and shoreline orientation. To accomplish this, we first reconstructed the approximate Pinheira shoreline at ~2.2 ka (2200 years ago or ~ 170 BCE) based on a single BFR that was preserved longitudinally across the strandplain (Figure SI1.1). This shoreline was chosen because it is one of few fully preserved BFR spanning the entire length of the Pinheira Strandplain (former coastline). Moreover, this time represents period during which Pinheira was characterized by a considerably different planform configuration as compared with more recent paleoshorelines.

The lateral contours and boundaries of the plain, such as the estimated locations of river mouths, were based on the evolutional model proposed by Hein *et al.* [1]. Other nearby features, such as the fronting bedrock headlands (today incorporated into the terrestrial strandplain) and Santa Catarina Island, were delimited from modern bathymetry/topography using the Paulo Lopes chart (IBGE). The area of these features was reduced through artificial flooding of the topographic surface to correlate with higher-than-present sea level at ~2.2 ka. Maximum Holocene sea level elevations were reached in Santa Catarina at approximately 5.8 ka, and has since been gradually fallen by *ca.* 0.6 m / 1000 years; by ~2.2 ka, sea level was ≤ 2 m above modern mean sea level[2,3].

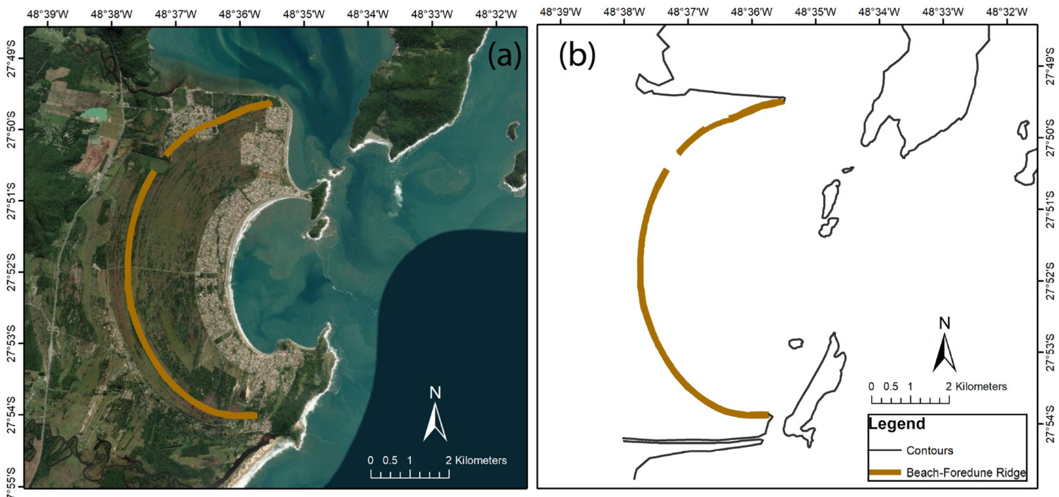

Figure SI1.1: Shoreline reconstruction for approximately ~2 ka. (a) Location of the mapped ~2.2 ka BFR (brown line). (b) Reconstructed ~2.2 ka-strandplain and bedrock headland coastlines.

Continental shelf bathymetry at ~2.2 ka is based on Brazilian nautical charts and General Bathymetric Chart of the Oceans (GEBCO) bathymetry gridded in the wave modelling software (SMC-Brazil). Modern shelf bathymetry was deepened by 2 m in accordance with higher-than present relative sea level at that time, and shelf deposition/erosion are assumed negligible. Holocene sediment along the Santa Catarina continental shelf is thin (< 10 m over 11,000 years)[4] and laterally homogenous. This allows for the assumption that changes in wave propagation in the last 3000 years responded primarily to changes in base level (sea level)[4] and independent allogenic climate forcings (Southern Atlantic wave climate). Shallow stratigraphic data show near-homogenous sub-parallel shoreface, foreshore, and foredune stratigraphy within progradational units across each cross-shore and alongshore transects throughout Pinheira Strandplain[1] (Figure SI1.2); Hein *et al.* [1] use this, and abundant sediment sampling, to infer a quasi-continuous supply of sediment from a distal source throughout the period of strandplain formation. From this it is assumed that refraction-diffraction patterns and the sediment types upon which incoming waves were acting did not change significantly through time as compared with to the modern beach. Therefore, the former beach (~2.2 ka) profile could be reconstructed within the wave modelling software based on modern Pinheira beach topographic-bathymetric profiles.

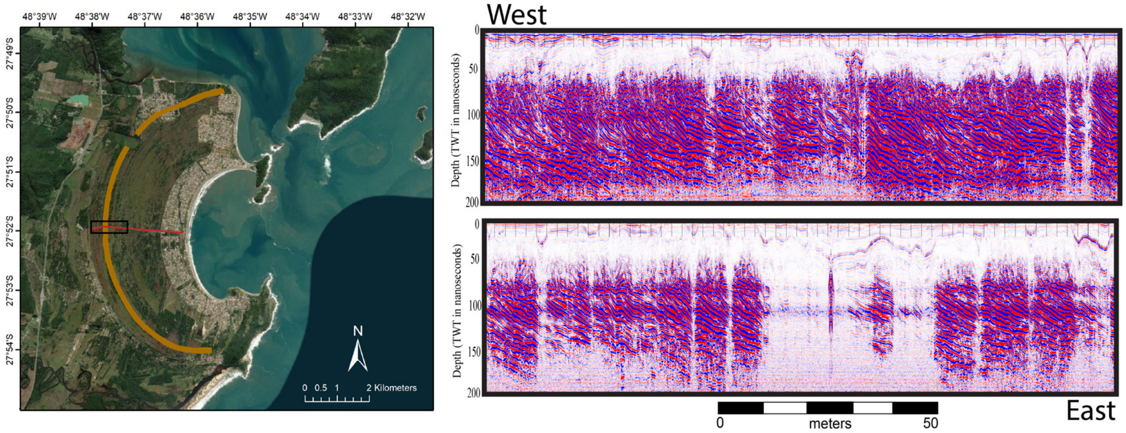

Figure SI1.2: Post-processed ground-penetrating radar transect from central Pinheira showing near-parallel shoreface and foreshore reflections. Data are from Hein *et al*. [1].

Section 2: Effective Mean Directional Wave Energy Flux

Wave propagation within the ~2.2 ka paleo-topographic/bathymetric grid was simulated in every 5° interval, from 45 to 180° N, totalizing 27 rounds for each environment, with equal propagation grids. Sixty years of hourly wave data was used with 100 cases selected for wave propagation from deep to shallow water. The mean directional wave energy flux (EF_θ) was reconstructed in shallow water in front of the BFR sets positions analysed at the modern beach.

The propagation of the wave climate in 5° directional sector bins allows for observation of wave transformation in response to obstacles (*e.g.*, bedrock headlands) and bathymetric variations within the study area. We observe that all waves from quadrants entering Pinheira Bay are altered through diffraction and refraction processes at the bay entrance, such that waves arrive at the central (BFR1) and central-north (BFR2) surf zone facing, respectively, east - 86 and 96°N for the modern beach (Figure SI2.1a) and 88 and 97°N for the ~2.2 ka beach (Figure SI2.1b), and east-southeast - 98 and 114°N for the modern beach (Figure SI2.1a) and 102 and 112°N for the ~2.2 ka beach (Figure SI2.1b). Despite applied changes in bathymetry, comparable results were found for both the modern and paleo bay.

Comparison of the direction of the EF_θ outside of the bay (modern and ~2.2 ka bathymetric depths of 15 and 17 m, respectively) and at the bay entrance between bedrock headlands (modern and ~2.2 ka bathymetric depths of 12 and 14 m, respectively) reveals minimal variation through time, confirming similarities in wave-refraction process on the adjacent continental shelf (Figure SI2.2). The EF_θ near the shoreline (5 m depth) also present similar direction ranges (Figure SI2.3). Differences are attributed to limitations of the ~2.2 ka bathymetric reconstruction, and to the longer profile for wave propagation within the paleo bay, allowing prolonged refraction process and consequent shifts in wave direction.

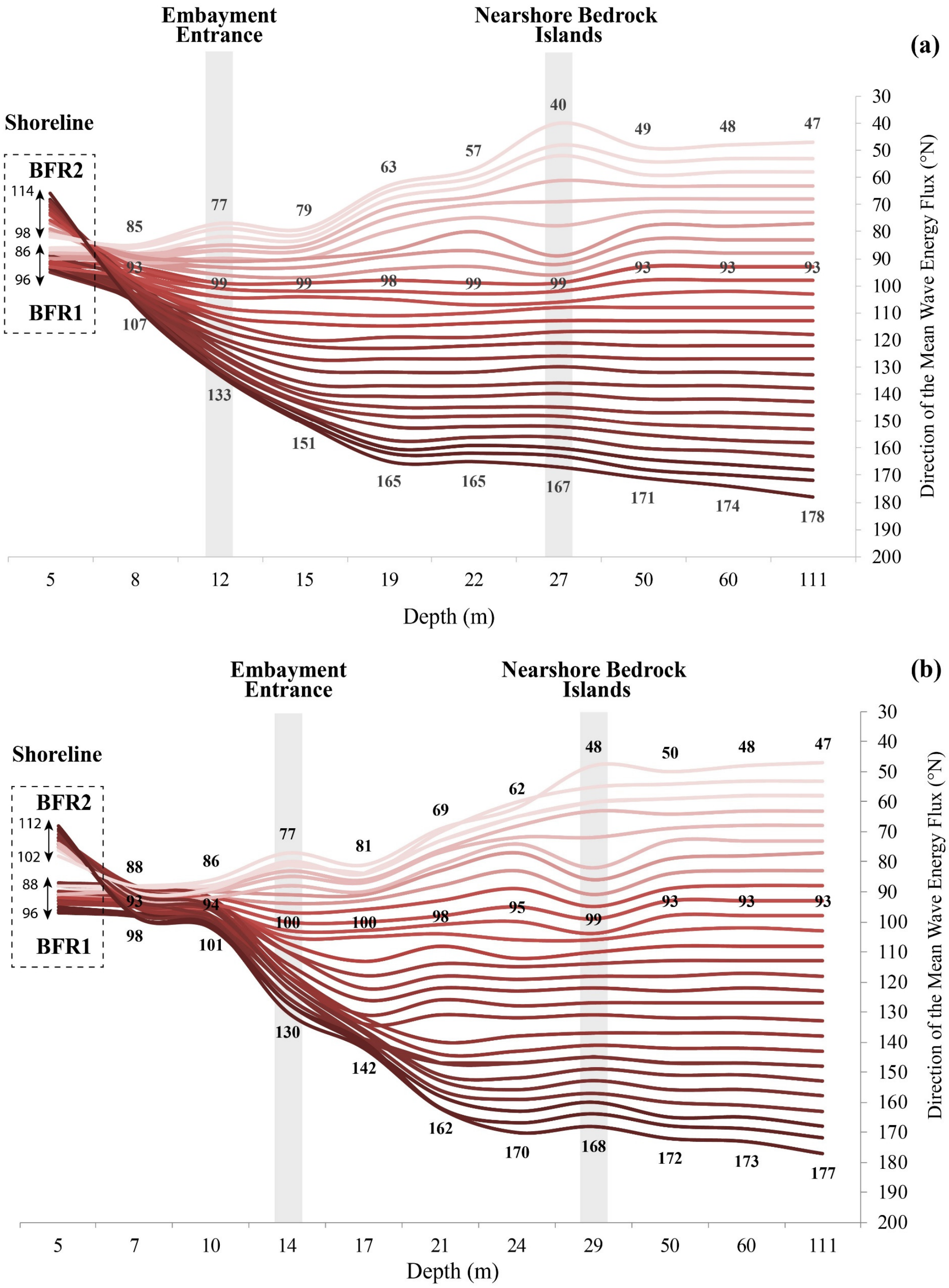

Figure SI2.1: Directional variation of the mean wave-energy flux distributed (vertical axis) along the interest points (horizontal axis, represented by their respective depths) for the (a) modern beach (upper graph) and for the (b) ~2.2 ka beach (lower graph). Lines indicate the propagation direction for each 5° sector bin, from 45-50° to 175-180°N.

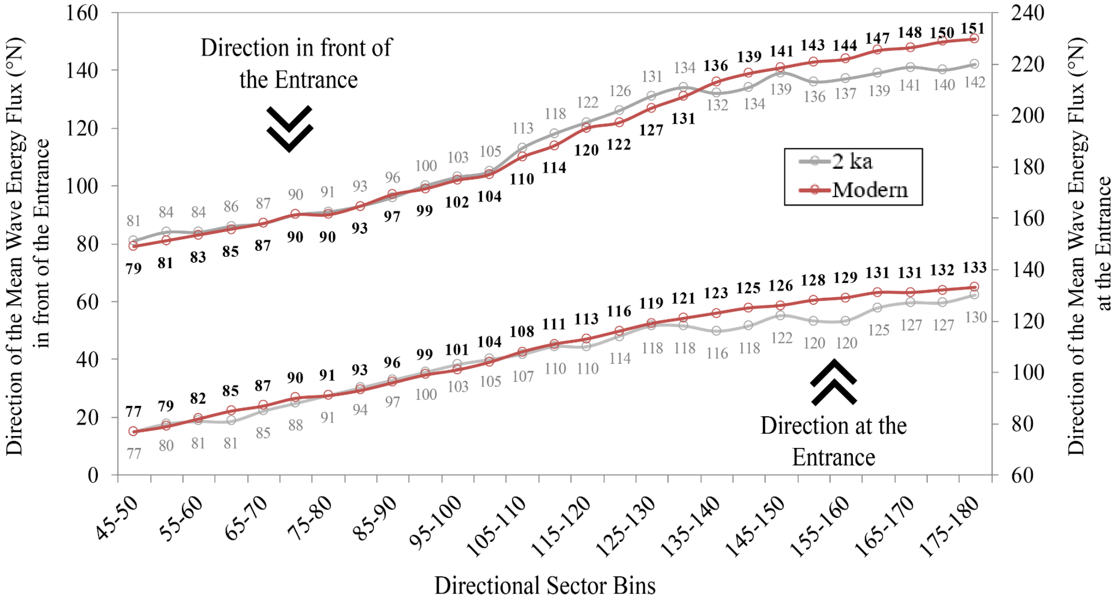

Figure SI2.2: Direction of the mean wave-energy flux seaward of (~15-17 m), and at (~12-14 m) the bay entrance, for each the modern (red circles, bolded values) and ~2.2 ka (gray circles and values) beaches.

Following propagation into the bay, NE waves (45–80°N) present low-energy values (<100 J·s^{-1}·m^{-1}) and are unlikely to transport significant volumes of sediment and modify the coastline; these are therefore treated as negligible. The potential transport of sediment obtained for this wave quadrant presented volumes of < 10,000 m^{3}·yr^{-1}. Waves from the eastern quadrant, especially those from between 80 and 105–110° N, encounter only minor obstructions along their propagation pathway. Although less energetic than those from the S-SE, the waves from this sector arrive with higher intensity (between ~130 and 600 J·s^{-1}·m^{-1}) (Figure SI2.4), and disperse throughout the length of the beach. Energy dissipation for easterly waves reaches up to only 50%, primarily associated with bottom friction.

Comparisons of the E (80–110° N) quadrant wave-energy flux intensity between each the modern and ~2.2 ka beach reveals similar energy dissipation outside of the embayment (Figure SI2.4), but lower EF_θ intensity reaching the shoreline in the ~2.2 ka simulations (maximum ~400 J·s^{-1}·m^{-1}) as compared with the modern (~575 J.s^{-1}.m^{-1}) (Figure SI2.3 and SI2.4). This is also attributed to greater extension of the inner bay profile, and consequently greater energy dissipation through bottom friction, as well as the presence of a larger bay perimeter for wave dispersion at ~2.2 ka (see "*3. Wave Propagation Charts*").

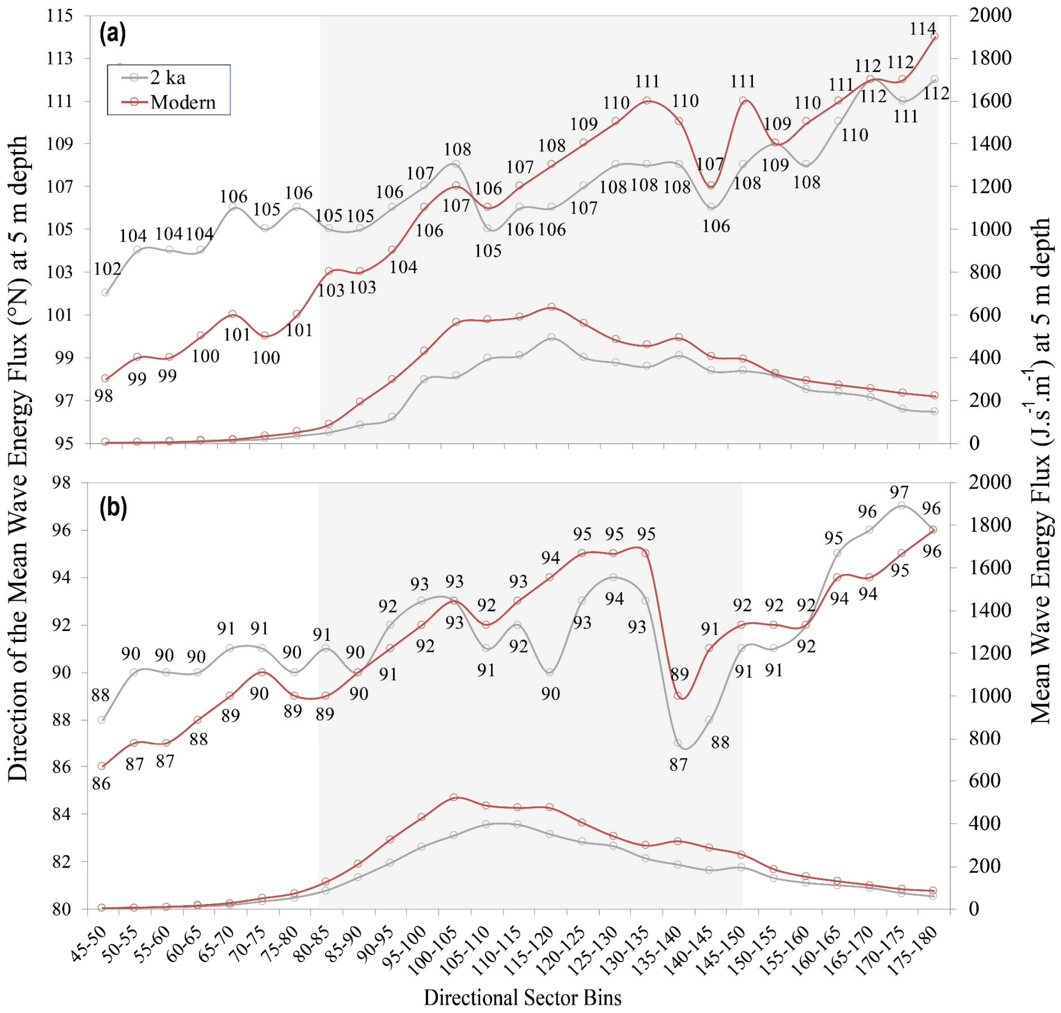

Figure SI2.3: Direction and wave-energy flux at 5 m depth for (a) BFR2 position and (b) BFR1 position. Direction of the mean wave-energy flux at 5 m depth for each the modern (red) and ~2.2 ka (grey) beaches is presented on top. Given below is mean wave-energy flux intensity for each the modern (red) and ~2.2 ka (gray) beaches. The gray shadow indicates the directional sector bins predominantly responsible for controlling shoreline orientation changes, as per model results.

Waves originating from the E-SE (110–135° N), vary from 20 to 40° between the origin and shoreline for both the modern and paleo beaches. However, directional changes outside of the bay are minimal (1–4°) (Figure SI2.1). Up to 90% of this directional shift occurs after waves have passed the headlands, which indicates that wave rotation is primarily associated with diffraction, and later refraction, within the bay. These E-SE waves have relatively high energy in deep water (maximum 1412 $J \cdot s^{-1} \cdot m^{-1}$) (Figure SI2.4), but once they reach the headlands, energy is transferred laterally along the wave crests and is dispersed into the shadow zone. This diffraction process decreases the energy

carried by the perpendicular crests moving to the shoreline by *ca.* 50–70% (maximum at BFR2 of 630 J·s^{-1}·m^{-1} and 475 J·s^{-1}·m^{-1} at BFR1 position) (Figure SI2.4).

(a)

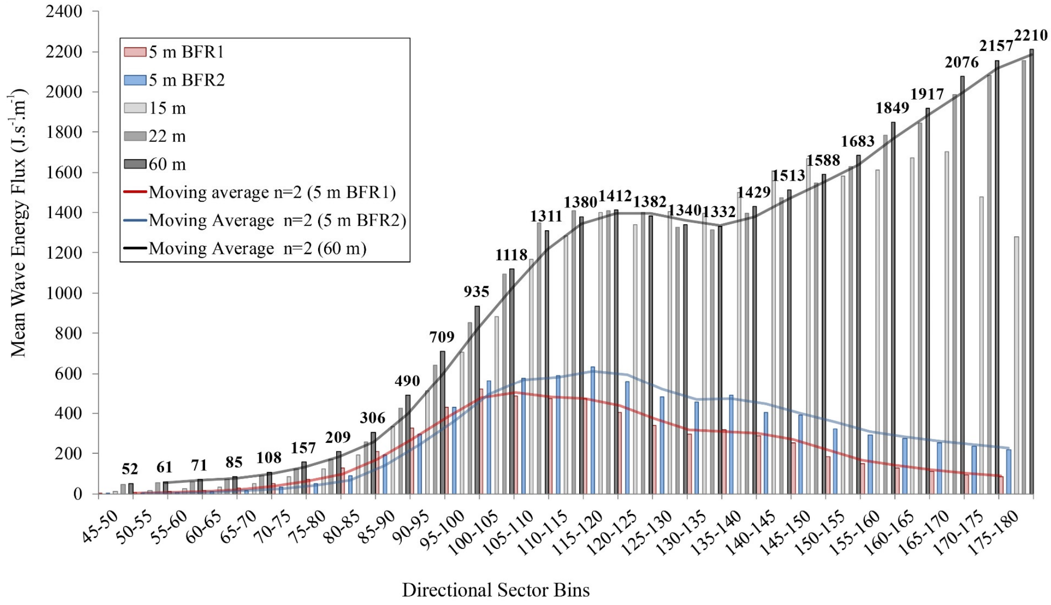

(b)

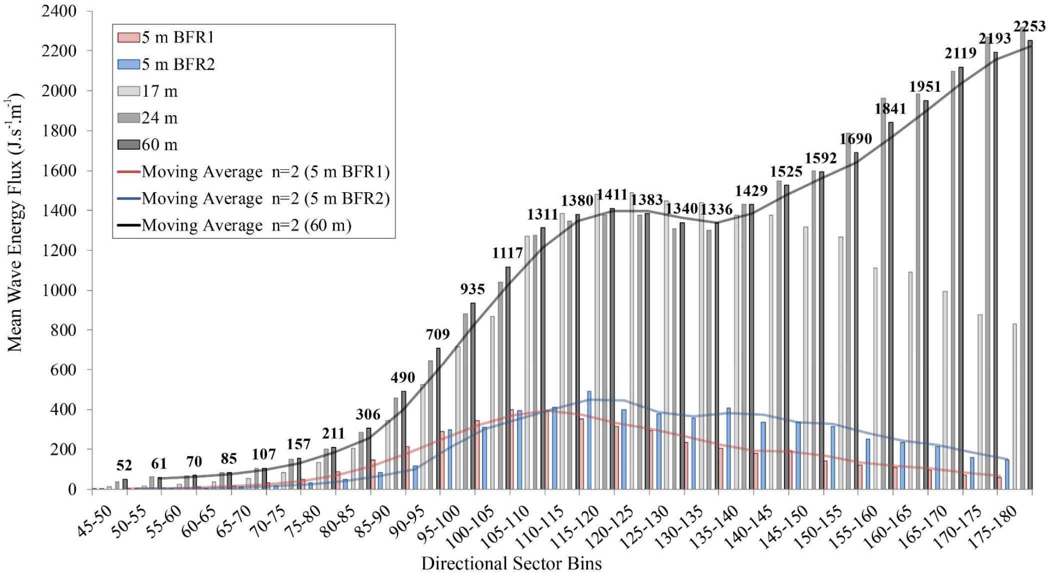

Figure SI2.4: Energy distribution along the propagation path (depth shown in the color legend), given in 5° directional sector bins (45–180° N), for each the (a) modern and (b) ~2.2 ka Pinheira Bay.

Among all wave directional sectors, the highest values of EF_θ inside the embayment (325 to 630 $J.s^{-1}.m^{-1}$) are from E-SSE waves (85–155° N). These directional quadrants are recognized as the effective mean directional wave-energy flux (effective EF_θ); that is, those associated with waves that are effectively able to perform morphological variations on the beach[5]. In the last decades, these directional sectors have represented about 78% of the wave climate inferred from the reanalysis series from the virtual offshore buoy (DOW point, 111 m depth) (Table SI2.1). In addition, BFR2 position also has wave energy reaching the nearshore with 220 $J.s^{-1}.m^{-1}$ from south quadrants (175-180º offshore), which allows the inclusion of these waves as morphological effective, reaching 91% of the offshore wave direction being represented by the BFR sets. Thus, these analyses together reveal that the Pinheira Strandplain preserves a record of significant long-term changes in predominant wave directions in the Subtropical South Atlantic.

This study presents a novel approach which takes advantage of the preserved sedimentary record to reconstruct paleo wave climate over multi-decadal to multi-centennial periods. Application of such an approach requires a semi-protected beach fed by near-homogenous sediments through time, which is sensitive to subtle changes in wave energy. Sequences of BFR preserved within embayed beaches are found world-wide[7], and therefore, given the right conditions (*e.g.*, well-preserved ridges, shoreline variability derived primarily from wave direction, etc.), they may serve as archives of long-term wave climate changes for the adjacent ocean basins.

Table SI2.1: Probability of wave direction and the mean ($\overline{Tp}$ and $\overline{Hs}$) and extreme (Tp_{12} and Hs_{12}) peak period and significant height for 96% of the directions of the virtual offshore buoy (DOW). Probability of occurrence of waves from NNE to W-SW is negligible and not presented in the table. Red highlighted values correspond to 91% (red highlighted) of the incident waves at the virtual offshore buoy (DOW) location.

Directions	Probability (%)	$\overline{Tp}$ (s)	Tp_{12} (s)	$\overline{Hs}$ (m)	Hs_{12} (m)
NE	0.01	7.8	11.1	1.5	3.7
ENE	0.03	8.0	11.6	1.3	3.3
**E**	**0.13**	**8.0**	**11.8**	**1.5**	**3.4**
**ESSE**	**0.23**	**7.7**	**12.7**	**1.6**	**3.8**
**SE**	**0.22**	**7.8**	**13.5**	**1.7**	**4.0**
**SSE**	**0.20**	**8.9**	**14.5**	**1.9**	**4.4**
**S**	**0.13**	**10.3**	**14.6**	**2.3**	**5.0**
SSW	0.01	9.1	13.5	2.0	4.3

Section 3: Wave Propagation Charts

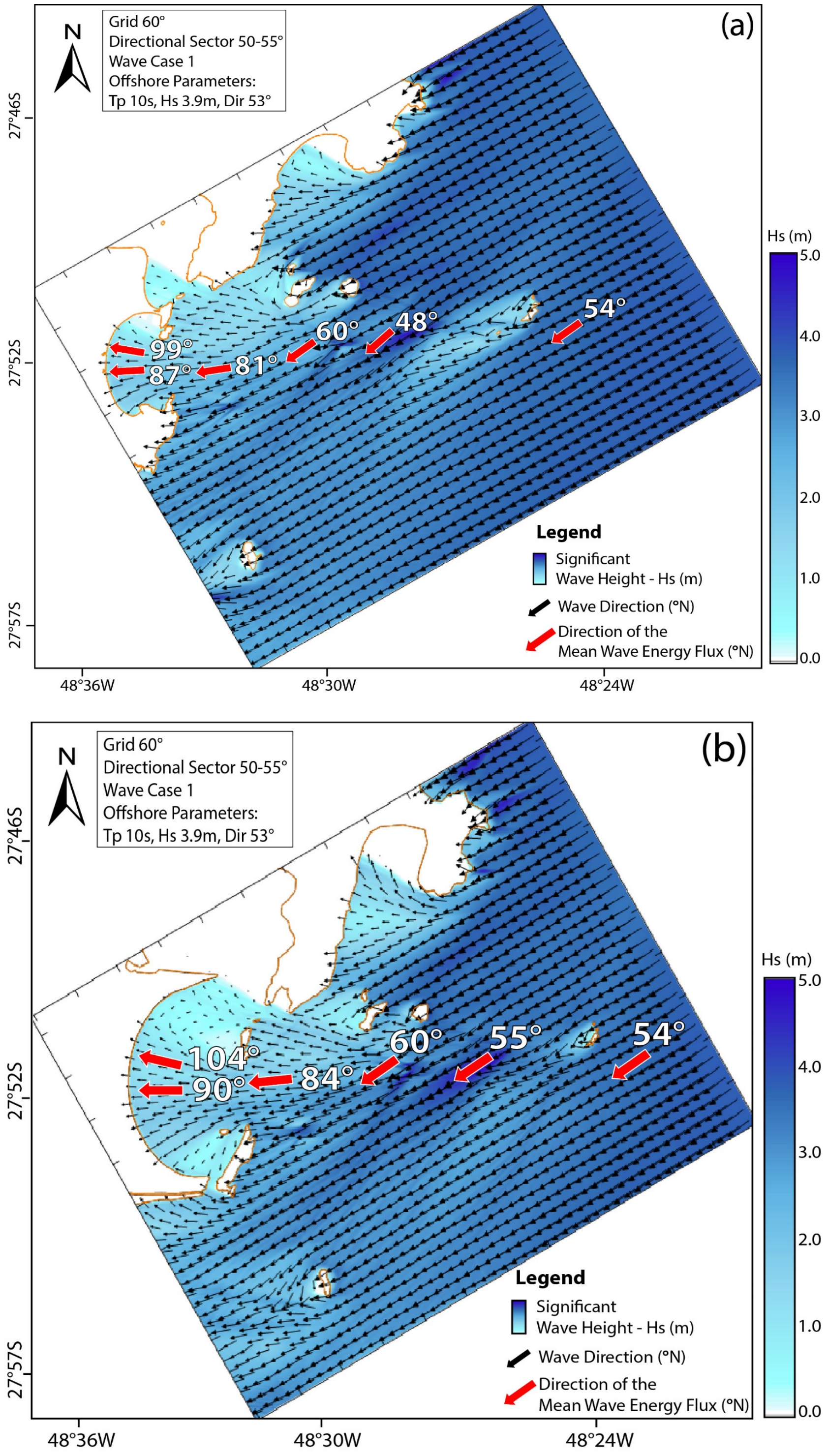

Figure SI3.1: Wave propagation maps showing wave height (m) (blue scale) and direction (black vectors) for each the modern (a) and 2.2 ka (b) Pinheira Bay and proximal shelf, for the Northeast quadrant. Red arrows indicate the direction of the mean wave-energy flux for the directional bin of 50–55° from 50 to 5 m depth (near shoreline).

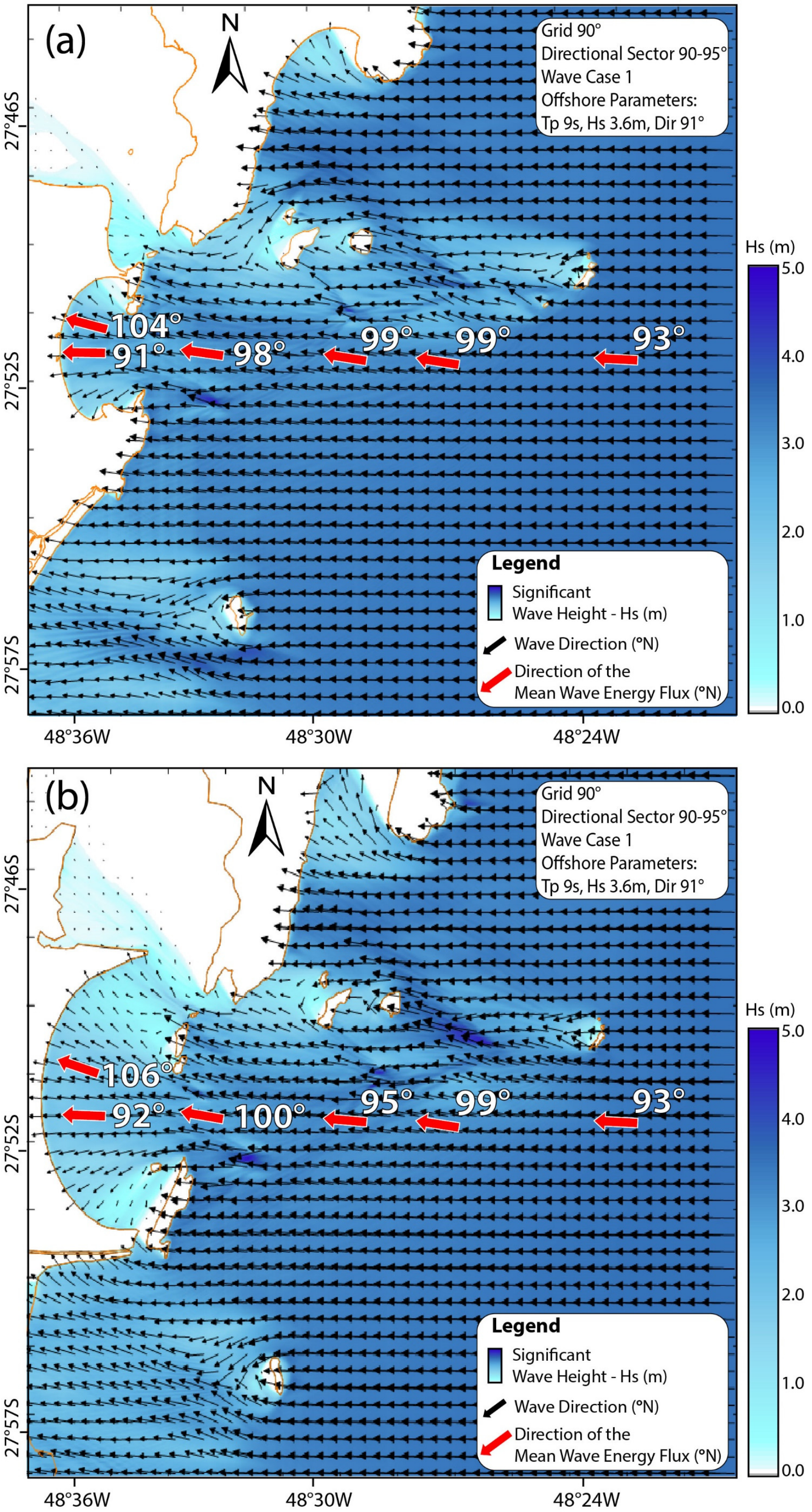

Figure SI3.2: Wave propagation maps showing wave height (m) (blue scale) and direction (black vectors) for each the modern (a) and ~2.2 ka (b) Pinheira Bay and proximal shelf, for the East quadrant. Red arrows indicate the direction of the mean wave-energy flux for the directional bin of 90–95° from 50 to 5 m depth (near shoreline).

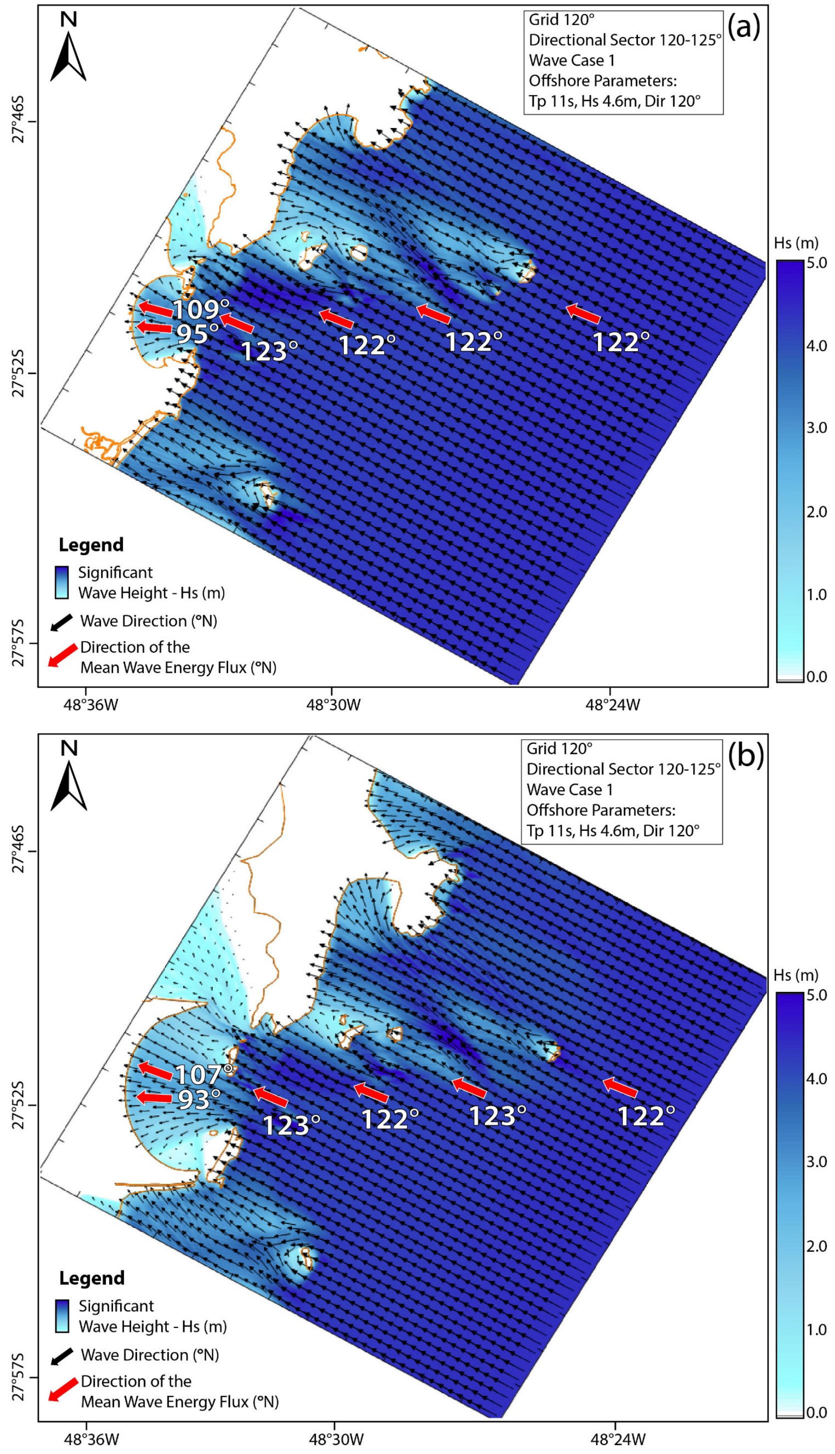

Figure SI3.3: Wave propagation maps showing wave height (m) (blue scale) and direction (black vectors) for each the modern (a) and ~2.2 ka (b) Pinheira Bay and proximal shelf, for the East-Southeast quadrant. Red arrows indicate the direction of the mean wave-energy flux for the directional bin of 120–125° from 50 to 5 m depth (near shoreline).

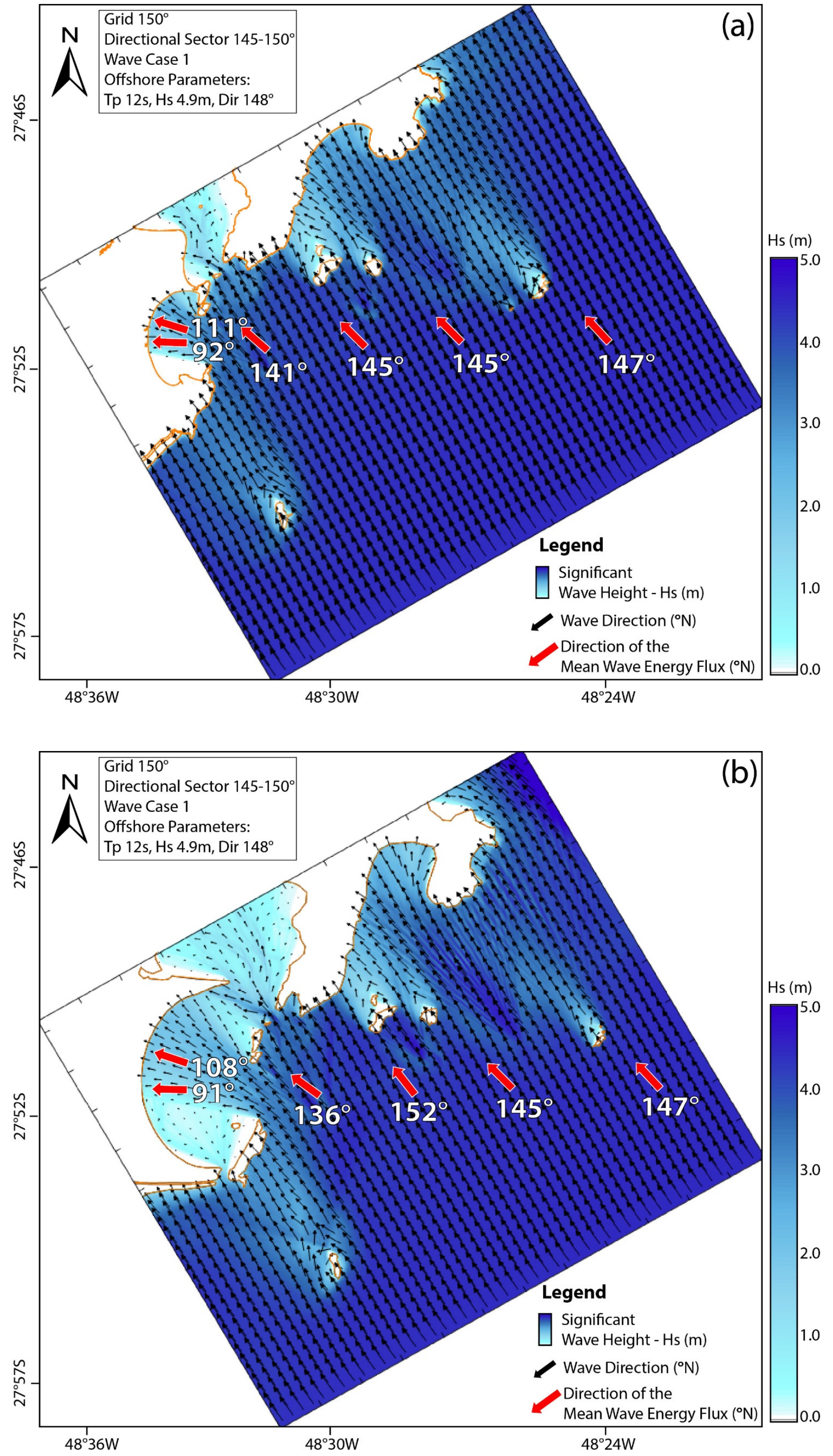

Figure SI3.4: Wave propagation maps showing wave height (m) (blue scale) and direction (black vectors) for each the modern (a) and ~2.2 ka (b) Pinheira Bay and proximal shelf, for the Southeast quadrant. Red arrows indicate the direction of the mean wave-energy flux for the directional bin of 145–150° from 50 to 5 m depth (near shoreline).

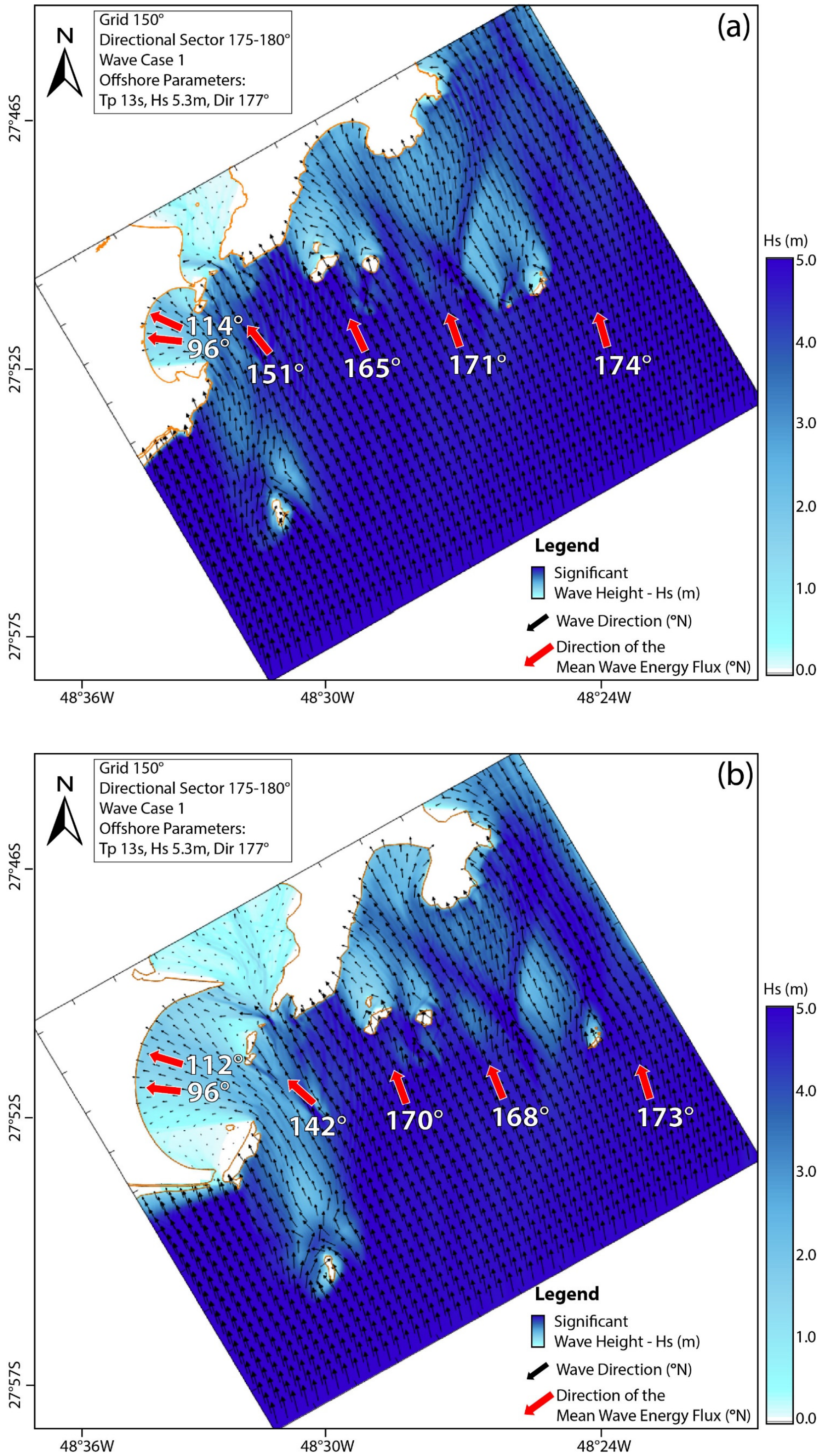

Figure SI3.5: Wave propagation maps showing wave height (m) (blue scale) and direction (black vectors) for each the modern (a) and ~2.2 ka (b) Pinheira Bay and proximal shelf, for the South quadrant. Red arrows indicate the direction of the mean wave-energy flux for the directional bin of 175–180° from 50 to 5 m depth (near shoreline).

Section 4: Principal Component of Sea-Level Pressure

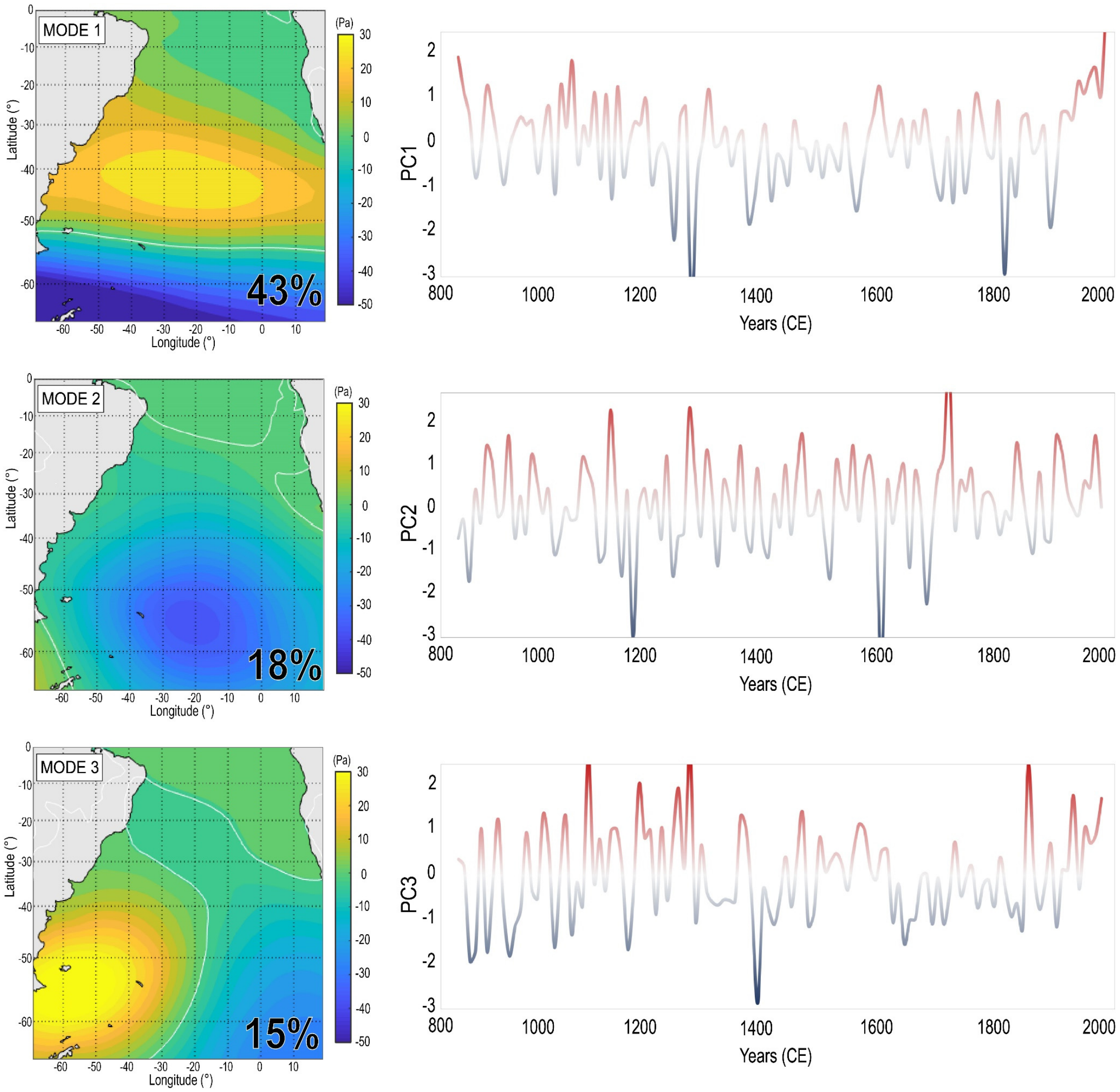

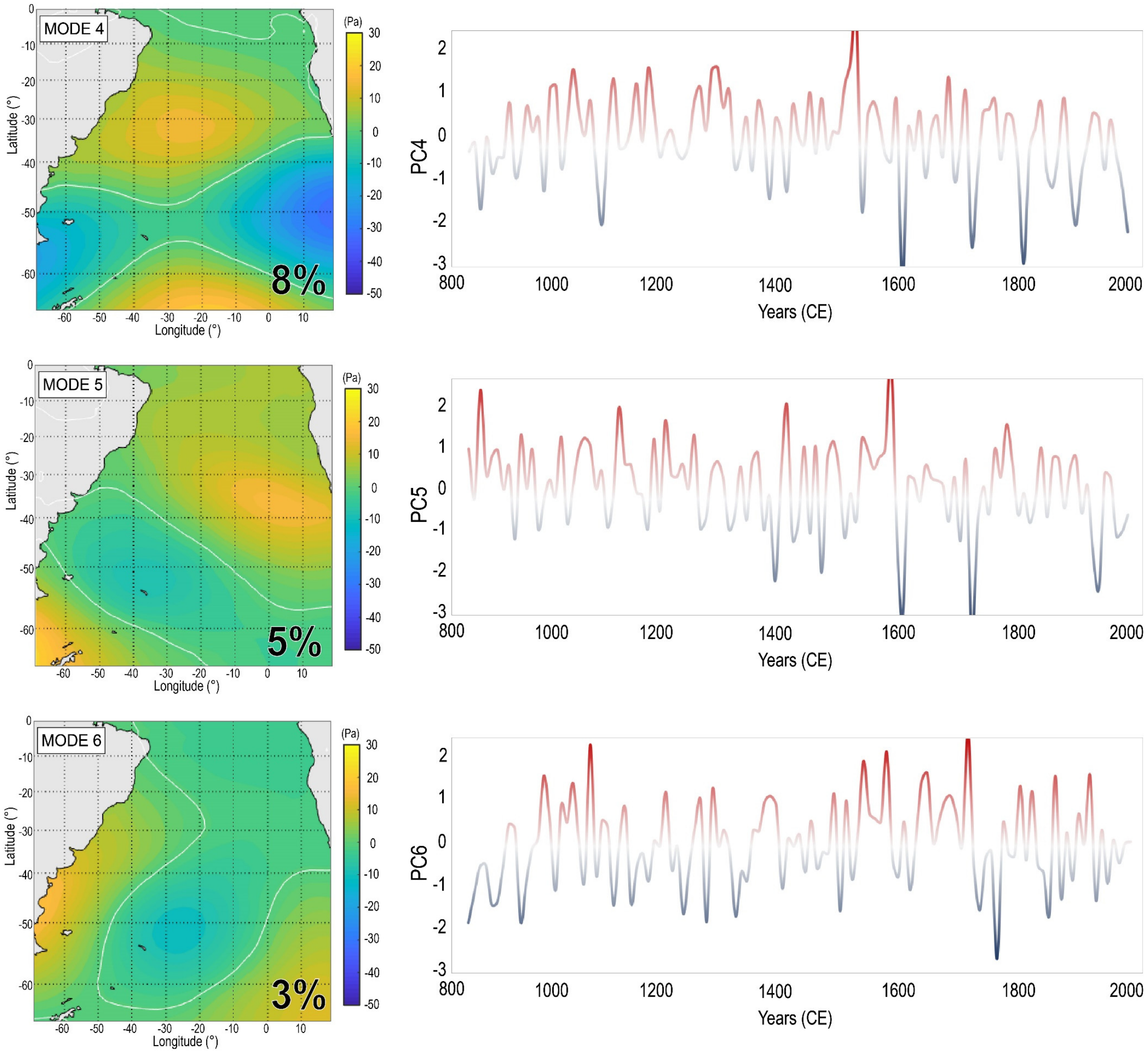

Figure SI4.1: Empirical Orthogonal Functions of sea level pressure (Pa), on the left, and the corresponding principal component time series of the six first EOFs modes. This analysis used sea level pressure over the South Atlantic basin between 850 and 2000 CE, from the NCAR/CESM Last Millennium Ensemble (LME).

REFERENCES

1. Hein, C.; Fitzgerald, D.; Cleary, W.; Albernaz, M.; Menezes J.T.; Klein, A.H.F. Evidence for a transgressive barrier within a regressive strandplain system: Implications for complex coastal response to environmental change. *Sedimentology* **59** (2013). doi: 10.1111/j.1365-3091.2012.01348.x
2. Angulo, R.J.; Lessa, G.C. The Brazilian sea level curves: a critical review with emphasis on the curves from Paranaguá and Cananéia regions. *Marine Geology* **140**, 141–166 (1997).
3. Angulo, R.J.; Lessa, G.C; Souza Filho, M.C. A critical review of mid- to late-Holocene sea-level fluctuations on the eastern Brazilian coastline. *Quaternary Science Reviews* **25**, 486 –506 (2006).
4. Cooper, J.A.G.; Meireles, R.P.; Green A.N.; Klein, A.H.F.; Toldo, E.E. Late Quaternary stratigraphic evolution of the inner continental shelf in response to sea-level change, Santa Catarina, Brazil. *Marine Geology* **397**, 1-14 (2018).
5. Reguero, B.G.; Méndez, F.J.; Losada, I.J. Variability of multivariate wave climate in Latin America and the Caribbean. *Global and Planetary Change* **100**, 70-84 (2013).
6. Elshinnawy, A.; Medina, R.; González, M. On the relation between the direction of the wave energy flux and the orientation of equilibrium beaches. *Coastal Engineering* **127**, 20-36 (2017).
7. Tamura, T. Beach ridges and prograded beach deposits as palaeoenvironment records. *Earth Science Reviews* **114**, 279-297 (2012).
